# Augmented secretary bird optimization algorithm for wireless sensor network deployment and engineering problem

**DOI:** 10.1371/journal.pone.0329705

**Published:** 2025-08-08

**Authors:** Qingwen Meng, Xi Kuang, Zihan Yu, Minghui He, Hanzhi Cui

**Affiliations:** 1 School of Computer, Electronics and Information, Guangxi University, Nanning, Guangxi, China; 2 International School of Business and Finance, Sun Yat-sen University, Guangzhou, China; 3 School of Material, Sun Yat-sen University, Guangzhou, China; 4 Virtual Reality (VR) Modern Industry College, Jiangxi University of Finance and Economics, Ganzhou, Jiangxi, China; 5 College of Computer Engineering, Qingdao City University, Shandong, China; Ningbo University, CHINA

## Abstract

This study develops an enhanced Secretary Bird Optimization Algorithm (ASBOA) based on the original Secretary Bird Optimization Algorithm (SBOA), aiming to further improve the solution accuracy and convergence speed for wireless sensor network (WSN) deployment and engineering optimization problems. Firstly, a differential collaborative search mechanism is introduced in the exploration phase to reduce the risk of the algorithm falling into local optima. Additionally, an optimal boundary control mechanism is employed to prevent ineffective exploration and enhance convergence speed. Simultaneously, an information retention control mechanism is utilized to update the population. This mechanism ensures that individuals that fail to update have a certain probability of being retained in the next generation population, while guaranteeing that the current global optimal solution remains unchanged, thereby accelerating the algorithm’s convergence. The ASBOA algorithm was evaluated using the CEC2017 and CEC2022 benchmark test functions and compared with other algorithms (such as PSO, GWO, DBO, and CPO). The results show that in the CEC2017 30-dimensional case, ASBOA performed best on 23 out of 30 functions; in the CEC2017 100-dimensional case, ASBOA performed best on 26 out of 30 functions; and in the CEC2022 20-dimensional case, it performed best on 9 out of 12 functions. Furthermore, the convergence curves and boxplot results indicate that ASBOA has faster convergence speed and robustness. Finally, ASBOA was applied to WSN problems and three engineering design problems (three-bar truss, tension/compression spring, and cantilever beam design). In the engineering problems, ASBOA consistently outperformed competing methods, while in the WSN deployment scenario, it achieved a coverage rate of 88.32%, an improvement of 1.12% over the standard SBOA. These results demonstrate that the proposed ASBOA has strong overall performance and significant potential in solving complex optimization problems. Although ASBOA performs well in these problems, its performance in high-dimensional multimodal problems and complex constrained optimization is unstable, and the introduced strategies add some complexity. Additionally, different parameter settings may lead to varying results, and the sensitivity of different problems to these parameters can also differ. It is necessary to adjust the settings according to the specific problem at hand in order to further refine and achieve a more stable version.

## 1. Introduction

With the continuous advancement of Wireless Sensor Network (WSN) technology and the expansion of its application scenarios, network coverage optimization has become a core research topic in the fields of the Internet of Things (IoT) and communication systems [1,2]. In modern smart cities, environmental monitoring, industrial automation, and similar domains, the deployment and management of WSNs play a critical role in determining network performance and efficiency. Optimizing network coverage not only enhances network reliability, ensuring stable and real-time data transmission, but also improves the accuracy and comprehensiveness of data collection. This is essential for achieving precise monitoring, timely responses, and efficient management [3,4].

By deploying sensor nodes strategically in space, the coverage area and signal quality of the sensor network can be significantly improved, thereby enhancing the precision and efficiency of task execution [5,6]. For instance, in environmental monitoring, an optimal node arrangement ensures broader area coverage and a more sensitive response to dynamic changes, improving the system’s real-time feedback capability. This, in turn, strengthens environmental monitoring and early warning mechanisms [7,8]. Moreover, optimized network layouts help reduce redundant data and communication conflicts, improving data collection accuracy and boosting the efficiency of decision-support systems. Rational network deployment not only increases resource utilization efficiency but also significantly extends the lifecycle of wireless sensor networks [9,10].

However, the optimization of WSNs faces numerous challenges. First, energy consumption of nodes is a critical issue, especially in large-scale networks where energy constraints often become a key factor affecting network performance. Additionally, environmental uncertainties, such as weather changes, obstacles, and node failures, further increase the complexity of network coverage optimization [11,12]. Traditional optimization methods, such as numerical optimization based on mathematical models and discrete search techniques, are effective in some simple scenarios. However, they often struggle when addressing complex global optimization problems. The high computational cost and limited adaptability of traditional approaches to dynamic environments create significant bottlenecks in practical applications [13].

Many researchers have conducted systematic studies on Wireless Sensor Networks (WSNs), leading to the development of various bio-inspired algorithms to optimize routing from member nodes to the sink node, with the goal of reducing energy consumption and prolonging network lifetime. For instance, Priyadarshi conducted a comprehensive study on WSN routing and clustering mechanisms, focusing on innovative optimization methods and offering a panoramic, in-depth analysis incorporating AI technologies [14]. Rawat proposed a cluster-based energy-efficient protocol for heterogeneous networks, which systematically utilizes sensor energy for cluster management, significantly enhancing network lifetime and reducing energy consumption [15]. To further extend network longevity, Rahul introduced a three-tier heterogeneous clustering scheme. This approach first classifies sensor nodes into three differentiated groups based on energy levels, then selects the optimal cluster head (CH) by considering energy thresholds and node efficiency metrics [16]. Intrusion Detection Systems (IDS) in WSNs largely rely on effective feature selection (FS) to improve performance. Nguyen proposed a novel method named Genetic Sacrificing Whale Optimization (GSWO) to overcome the limitations of traditional approaches [17]. Priyadarshi also introduced an efficient cluster head formation technique that significantly optimizes energy utilization, thereby achieving superior network lifetime performance [18]. Addressing the critical issue of limited battery life in WSN nodes, Rahul and colleagues developed a novel and efficient cluster head selection mechanism—an Energy-Dependent Clustering Framework (EDCF) for heterogeneous WSNs—designed to significantly extend network lifespan [19]. To tackle challenges such as high deployment costs and insufficient effective coverage in WSNs, Qu et al. proposed a coverage optimization method based on an Improved Multi-Strategy Grey Wolf Optimizer (IGWO-MS) [20]. Bharat Gupta and colleagues suggested enhancing network coverage through slight node repositioning: they accurately identified blind spots within the monitored area and calculated optimal new positions for mobile nodes [21]. Raj Vikram introduced an Improved Triangular-Based Localization Scheme (MTBLS) aimed at enhancing the performance of traditional Midpoint-Based Localization Scheme (MBLS) and Triangular-Based Localization Scheme (TBLS), thereby better meeting the communication needs of intelligent distribution automation systems [22].

To address these challenges, metaheuristic algorithms have emerged. These algorithms, by simulating processes such as biological evolution, collective behavior, or physical phenomena in nature, exhibit powerful global search capabilities [23,24]. Compared to traditional optimization methods, metaheuristic algorithms are more effective in handling nonlinear and high-dimensional optimization problems, making them particularly suitable for optimization tasks in complex and uncertain environments [25]. Piyush proposed a clustering protocol called the Efficient Cluster Head Selection Scheme (ECSS), which enhances overall network lifetime and performance by preferentially selecting high energy-efficient cluster heads (CHs) [26]. Rahul Priyadarshi introduced a cube-based three-dimensional coverage model and deployment framework. By establishing a quantitative relationship between the sensor’s radius and its coverage area, the model calculates the minimum number of nodes required to achieve full coverage [27]. In WSN coverage optimization, metaheuristic algorithms such as Genetic Algorithm (GA) [28], Particle Swarm Optimization (PSO) [29], and Ant Colony Optimization (ACO) [30] have been widely applied and have achieved significant results [24,31]. By simulating the adaptive evolution process of biological populations, these algorithms not only enable global search but also facilitate rapid escape from local optima. As a result, they are better able to tackle the various complex problems in WSNs, offering high optimization efficiency and strong practical value [32]. Furthermore, to address the sensitivity of current algorithms to hyperparameters, Blan investigated adaptive control and self-configuration mechanisms to enhance robustness and reduce reliance on manual parameter tuning [33].

Generally, metaheuristic algorithms can be categorized into four types [34,35]: Evolutionary Algorithms (EA), Physics-based Algorithms (PhA), Human-based Algorithms (HB), and Swarm Intelligence (SI)-based Algorithms. Evolutionary Algorithms include Differential Evolution (DE) [36] and Genetic Algorithms (GA) [28], among others. Physics-based Algorithms include Simulated Annealing (SA) [37], and Gravitational Search Algorithm (GSA) [38]. Human-based Algorithms include Teaching–Learning-based Optimization Algorithm (TLBO) [39], Social Evolution and Learning Optimization (SELO) [40], Love Evolution Algorithm (LEA) [41], and Gold Rush Optimization algorithm (GRO) [42], among others. Swarm Intelligence-based Algorithms include Bat Algorithm (BA) [43], Grey Wolf Optimizer (GWO) [44], Harris Hawks Optimization (HHO) [45], Golden Jackal Optimization (GJO) [46], Quantum Avian Navigation Algorithm (QANA) [47], Black Widow Optimization (BWO) [48], Red-beaked Magpie Optimization (RBMO) [49], Golden Eagle Optimizer (GEO) [50],Genghis Khan shark optimizer(GKSO) [51], and Goose Optimization Algorithm (GOOSE) [52], among others. These organisms demonstrate remarkable problem-solving capabilities through decentralized, self-organized interactions among individuals. Researchers in the field of Swarm Intelligence (SI) aim to understand and replicate such characteristics in artificial systems, thereby developing algorithms and methods capable of effectively addressing a wide range of optimization challenges. The fundamental concept of SI systems is based on emergent intelligence, where simple entities following local rules collectively exhibit complex global behaviors. This provides an effective approach for solving various complex optimization problems [53].

Secretary Bird Optimization Algorithm(SBOA) [54] is a recently proposed swarm intelligence-based metaheuristic algorithm. Compared to traditional optimization algorithms, SBOA has significant advantages in optimization performance, with its simple structure and efficient solving ability standing out. However, like most metaheuristic algorithms, SBOA still faces potential challenges such as slow convergence speed and the risk of falling into local optima. To mitigate the risk of SBOA getting trapped in local optima while improving its convergence speed, this paper proposes three improvement strategies: the Differential Cooperative Search Mechanism, the Optimal Boundary Control Mechanism, and the Information Retention Control Mechanism. Based on these strategies, a new Augmented Secretary Bird Optimization Algorithm (ASBOA) is introduced, which is applied to Wireless Sensor Network (WSN) problems and engineering problems. The main contributions of this paper are as follows:

(1) Three improvement strategies are proposed and applied to SBOA, resulting in an enhanced version called ASBOA;
(2) Comparative experiments are conducted with eight benchmark algorithms using the CEC2017 benchmark suite, verifying the optimization performance of ASBOA;
(3) ASBOA is applied to the optimization of Wireless Sensor Network layout problems, further demonstrating its effectiveness in solving real-world engineering problems.

The remaining sections of this paper are organized as follows: Section 2 provides an introduction and modeling of the Wireless Sensor Network (WSN) model. Section 3 discusses the standard SBOA algorithm and the improved ASBOA version. Section 4 presents a detailed analysis of the experimental results. Section 5 offers a summary of the paper and discusses future prospects.

## 2. WSN mathematical model

### 2.1 Basic concepts of WSN

**Fig 1:**
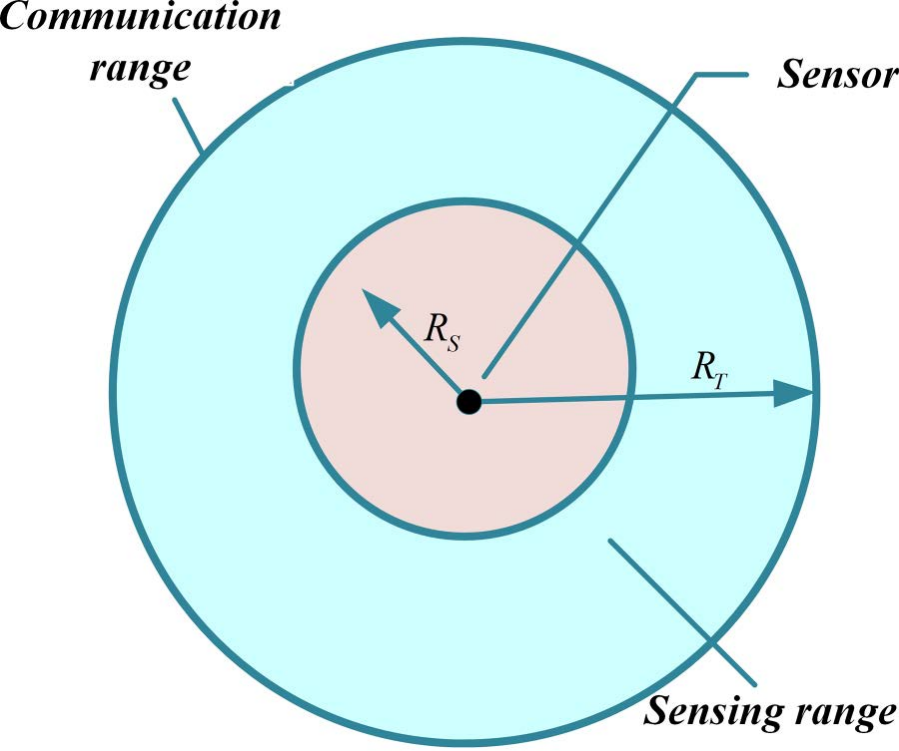
Sensor node perception framework.

**Fig 2:**
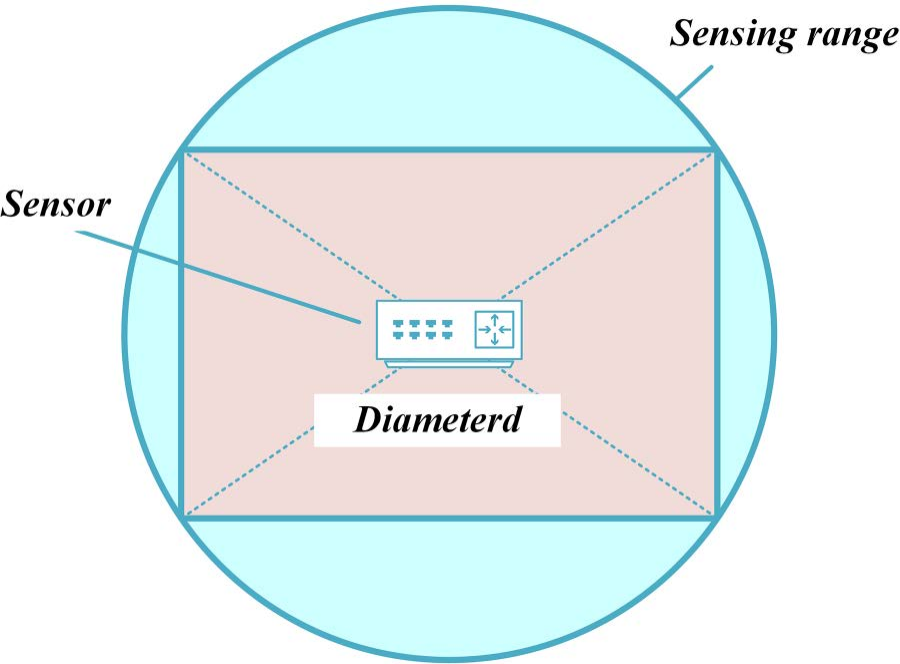
The sensing area of sensor in terms of square.

We examine the sensor’s sensing model depicted in Fig 1, where two concentric circles are drawn around the sensor. The inner circle represents the sensing area, denoted as $R_{S}$, which corresponds to the radius of the disk. The outer circle indicates the communication range, represented by $R_{T}$. We assume that $R_{T}=2\times R_{S}$. It has been demonstrated that if the communication range is at least twice the sensing range, this condition is adequate to guarantee complete coverage of the convex region, thereby ensuring connectivity among active nodes [11]. However, this assumption of omnidirectional sensing capability does not apply to certain types of sensor nodes, such as cameras or ultrasonic sensors with directional sensing areas. As shown in Fig 2, if the circular sensing area is transformed into a square, the circle’s diameter becomes the diagonal of the square [11,31,55].

The sensor is powered by a battery, so it can only operate for a limited period. Therefore, energy-efficient and coverage protocols are required to extend the battery’s lifetime. The probability of event detection is inversely related to the Euclidean distance between the sensor and the event. In a Wireless Sensor Network (WSN), there are two sensor detection models to identify effective coverage. These are the binary detection model, assuming no uncertainty, and the probability perception model with random detection. The probabilistic coverage model offers a more accurate representation of real sensor performance in an environment. On the other hand, the binary perception model, which is the most basic and commonly studied coverage model, assumes that a sensor detects all events within its sensing range [23]. In this paper, we adopt the binary detection model, as expressed in Equation (1).


$$\begin{array}{c} \begin{array}{c} P_{d}\text{ = }\left\{ \begin{array}{c} \text{1},\;if\text{\textbackslash{} }\left\|p_{e}-p_{s}\right\|\text{ < }R_{s}\\ \text{0,}\text{\textbackslash{} }\text{\textbackslash{} \textbackslash{} \textbackslash{} \textbackslash{} \textbackslash{} \textbackslash{} \textbackslash{} \textbackslash{} }otherwise\text{.} \end{array}\right.\; \end{array} \end{array}$$ (1)

Here, $P_{d}$ is the probability that an event occurring at position $p_{e}$ is detected by a sensor located at position $p_{s}$. $\left\|p_{e}-p_{s}\right\|$ represents the Euclidean distance between the sensor and the event.

### 2.2 Constraints and objective functions of WSN

The objective function proposed in this study primarily considers coverage. First, we assume the sensor nodes are denoted as $p_{i\text{,}j}$, and the deployment space is $s_{i\text{,}j}$. The coverage radius of the sensor is $R_{T}$, and the covered points are ${Cover}_{i\text{,}j}$. The coverage equation is given by Equation (2).


$$\begin{array}{c} {Cover}_{i\text{,}j}\text{ = }\left\{ \begin{array}{c} \text{1},\;if\text{\textbackslash{} }d\left(p_{i\text{,}j},\;s_{i\text{,}j}\right)\leq \;R_{T}\\ \;\;\;\;\;\;\;\;\;\;\;\text{0,}\text{\textbackslash{} }\text{\textbackslash{} \textbackslash{} \textbackslash{} \textbackslash{} \textbackslash{} \textbackslash{} \textbackslash{} \textbackslash{} }otherwise\text{.} \end{array}\right.\; \end{array}$$ (2)

Here, $d\left(p_{i\text{,}j},\;s_{i\text{,}j}\right)$ denotes the Euclidean distance between the sensor $p_{i\text{,}j}$ to the point $s_{i\text{,}j}$, which is calculated using Equation (3).


$$\begin{array}{c} d\left(p_{i\text{,}j},\;s_{i\text{,}j}\right)\text{ = }{\left\|s_{i\text{,}j}\text{ - }p_{i\text{,}j}\right\|}_{\text{2}} \end{array}$$ (3)

Therefore, the coverage probability of the deployment area is as follows:


$$\begin{array}{c} Coverage\text{\textbackslash{} }probability\text{ = }\frac{\sum_{i\text{ = 1}}^{n}\sum_{j\text{ = 1}}^{m}{Cover}_{i\text{,}j}\text{\textbackslash{} }\text{\textbackslash{} }}{m\times n} \end{array}$$ (4)

In this equation, m and n are the length and width of the deployment space. The objective of this problem is to maximize the network coverage, which is a maximization problem. Therefore, its fitness function F is:


$$\begin{array}{c} F\text{ = }Coverage\text{\textbackslash{} }probability\text{ = }\frac{\sum_{i\text{ = 1}}^{n}\sum_{j\text{ = 1}}^{m}{Cover}_{i\text{,}j}\text{\textbackslash{} }\text{\textbackslash{} }}{m\times n} \end{array}$$ (5)

## 3. Secretary bird optimization algorithm and Augmented secretary bird optimization algorithm

### 3.1 Secretary bird optimization algorithm

#### 3.1.1 Inspiration of Secretary bird optimization algorithm.

The inspiration for the Secretary Bird Optimization Algorithm (SBOA) comes from the natural survival behavior of the secretary bird, primarily simulating its hunting strategy and escape behavior from predators or enemies. The three stages of the secretary bird’s hunting behavior correspond to the exploration phase of the algorithm, while the two strategies for escaping predators and enemies correspond to the exploitation phase of the algorithm [56,57].

#### 3.1.2 Mathematical model of Secretary bird optimization algorithm.

The mathematical model of the Secretary Bird Optimization Algorithm (SBOA) is established in three stages: the initialization phase, the exploration phase, and the exploitation phase. The mathematical models for each stage are presented as follows:

**Initialization Phase:** To solve the optimization problem, an initial solution for the search needs to be determined, which is initialized using the following equation:


$$\begin{array}{c} x_{i\text{,}j}\text{ = }\left({ub}_{j}\text{ - }{lb}_{j}\right)\times r_{1}\text{ + }{lb}_{j} \end{array}$$ (6)

Where, $x_{i,j}$ represents the initial value of the $i^{th}$ candidate solution’s $j^{th}$ decision variable; ${ub}_{j}$ and ${lb}_{j}$ represent the upper and lower bounds, respectively, and $r_{1}$ is a random number in the range (0, 1).

**Exploration Phase:** This phase is divided into three stages: searching for prey $\left(iter<\frac{1}{3}T_{max}\right)$,exhausting prey $\left(\frac{1}{3}T_{max}<iter<\frac{2}{3}T_{max}\right)$,and attacking prey $\left(iter>\frac{2}{3}T_{max}\right)$. In the “searching for prey” stage, the secretary bird looks for potential prey. Once prey is identified, it moves into the “exhausting prey” stage, where it consumes the prey’s energy. The bird, with sharp judgment of the prey’s movements, leisurely wanders, jumps, and provokes near the prey, gradually depleting the prey’s energy. When the prey’s stamina is nearly exhausted, the bird attacks. This process is modeled using equations (7) and (8) [54].


$$\begin{array}{c} x_{i,j}^{new1}=\left\{ \;\;\;\;\begin{array}{c} {\;x}_{i,j}+r_{2}\times {(x}_{R_{1}}-x_{R_{2}}),\;if\;iter<\frac{1}{3}T_{max}\\ x_{best}+exp\left({\left(\frac{iter}{T_{max}}\right)}^{4}\right)\times (RB-0.5)\times \left(x_{best}-x_{i,j}\right),\;if\;\frac{1}{3}T_{max}\leq iter<\frac{2}{3}T_{max}\\ \;\;\;\;\;\;\;\;\;\;\;\;{\;x}_{best}+{\left(1-\frac{iter}{T_{max}}\right)}^{\left(2\times \frac{iter}{T}\right)}\times x_{i,j}\times RL,\;else \end{array}\right.\; \end{array}$$ (7)

$$\begin{array}{c} X_{i}=\left\{ \;\;\;\begin{array}{c} \;{\;X}_{i}^{new1},\;if\;F_{i}^{new1}<F_{i}\\ X_{i\;}\;,else \end{array}\right.\; \end{array}$$ (8)

In this context, iter represents the current iteration number, and $T_{max}$ denotes the maximum number of iterations. ${\;X}_{i}^{new1}$ represents the new state of the $i^{th}$ secretary bird in the first stage. $x_{R1}$ and $x_{R2}$ are the random candidate solutions in the first-stage iteration, and $r_{2}$ is a random array of dimensions 1×dim generated from the interval [0, 1]. $x_{i,j}^{new1}$ represents the position information of the $j^{th}$ dimension of the solution. $F_{i}^{new1}$ indicates the fitness value of the objective function for that solution. RB represents an array of dimensions 1×dim randomly generated from the standard normal distribution (mean = 0, standard deviation = 1). $x_{best}$ denotes the global best solution, RL refers to the Lévy flight function, which is calculated using equation (9).


$$\begin{array}{c} \left\{ \;\;\;\begin{array}{c} RL=0.5\times Levy(dim)\;\\ \;\;\;\;\;\;Levy(dim)=0.01\times \frac{u\times \sigma }{|v{|}^{\frac{1}{\eta }}}\;\\ \;\;\;\;\;\;\;\;\;\;\;\;\;\;\;\;\;\;\sigma ={\left(\frac{𝛤(1+\eta )\times sin\left(\frac{\pi \times \eta }{2}\right)}{𝛤\left(\frac{1+\eta }{2}\right)\times \eta \times 2\left(\frac{\eta -1}{2}\right)}\right)}^{\frac{1}{\eta }} \end{array}\right.\; \end{array}$$ (9)

In this equation, σ and η are fixed constants with a value of 1.5. u and v are random numbers generated within the interval [0, 1], and 𝛤 represents the Gamma function.

Exploitation Phase**:** During this phase, the secretary bird may encounter attacks from predators or competitors trying to steal its food. It is highly intelligent and typically adopts various evasive strategies to protect itself or its food. These strategies are primarily divided into two types: one is to escape by flying or running $\left(S_{1}\right)$, and the other is camouflage, where the secretary bird uses environmental colors or structures to blend in and make itself harder for predators to detect $\left(S_{2}\right)$[54].This process is modeled using equations (10) and (11).


$$\begin{array}{c} {\;x}_{i,j}^{new2}=\left\{ \begin{array}{c} \;{S_{1}\;\;:\;x}_{best}+(2\times RB-1)\times {\left(1-\frac{iter}{T_{max}}\right)}^{2}\times x_{i,j}\;,\;if\;q<r_{3}\\ \;\;\;S_{2}\;\;:{\;x}_{i,j}+r_{4}\times \left(x_{rand}-l\times x_{i,j}\right)\;,\;else \end{array}\right.\; \end{array}$$ (10)

$$\begin{array}{c} X_{i}=\left\{ \;\;\;\begin{array}{c} \;{\;X}_{i}^{new2},\;if\;F_{i}^{new2}<F_{i}\\ X_{i\;}\;,else \end{array}\right.\; \end{array}$$ (11)

In this context, q=0.5, $r_{3}$ and $r_{4}$ represent arrays of dimensions (1×dim) randomly generated from a normal distribution. $x_{rand}$ is the random candidate solution for the current iteration, $x_{best}$ represents the global best solution, and l is a randomly selected integer, either 1 or 2.

### 3.2 Augmented secretary bird optimization algorithm

#### 3.2.1 Differential cooperative search mechanism.

Faced with the differences among individuals, understanding the underlying causes of these variations, and learning from them, individual growth can be greatly enhanced, leading to a faster convergence of the algorithm. However, in the attack prey stage of the SBOA’s exploration phase, relying solely on the information from the best individual can lead to the algorithm getting stuck in local optima, and fail to further explore the solution space in search of better solutions. To address this issue, a differential collaborative search mechanism has been introduced in SBOA’s attack prey 1 to facilitate the calculation process. This allows every individual to potentially participate in the computation, ensuring that valuable information surrounding the global best region is preserved [58,59]. In each gap, individuals from adjacent levels consistently engage in the operations, thereby introducing diverse evolutionary information to the population. However, the last two gaps do not adhere to this pattern, intentionally introducing an element of uncertainty into the evolutionary process. Specifically, the learning operator in ASBOA utilizes four gaps designed to approximate the fitness landscape. These gaps are outlined in Equation (12).


$$\begin{array}{c} \left\{ \;\;\;\begin{array}{c} {Gap}_{1}=x_{best}-x_{better}\;\\ {Gap}_{2}=x_{best}-x_{worse}\;\\ {Gap}_{3}=x_{better}-x_{worse}\\ {Gap}_{4}=x_{l1}-x_{l2}\; \end{array}\right.\; \end{array}$$ (12)

Here, $x_{best}$ represents the global best solution, while $x_{better}$ denotes the second-best solution, often referred to as the elite solution. $x_{worse}$ represents the worst solution, and $x_{l1}$ and $x_{l2}$ are random individuals.${Gap}_{k}(k=1,2,3,4)$ represents the gap between two individuals, allowing the learner to fully comprehend the differences between them and leverage these differences for improvement.

To account for this variability, the learning factor LF is introduced for each of the four differential measures. For the $i^{th}$ individual, it influences the $k^{th}$ group gap, and is modeled as follows (equation (13)):


$$\begin{array}{c} {LF}_{k}=\frac{\left\|{Gap}_{k}\right\|}{\sum_{k=1}^{4}\left\|{Gap}_{k}\right\|},\;(k=1,2,3,4) \end{array}$$ (13)

Where ${LF}_{k}$ represents the normalized ratio of the Euclidean distance for the $k^{th}$ group gap $\left({Gap}_{k}\right)$, with a range of [0, 1]. When the $k^{th}$ group is larger, ${LF}_{k}$ also increases, indicating that the $i^{th}$ individual will learn more from the $k^{th}$ gap.

During the search process, secretary birds at different positions have different perspectives on themselves. The $i^{th}$ secretary bird uses ${SF}_{i}$ to evaluate its own acceptable range of knowledge. A larger ${SF}_{i}$ indicates that the individual needs to learn more to improve itself. ${SF}_{i}$ is modeled using equation (14):


$$\begin{array}{c} {SF}_{i}=\frac{{Fitness}_{i}}{{Fitness}_{max}} \end{array}$$ (14)

Where ${Fitness}_{i}$ represents the fitness value of the individual, and ${Fitness}_{max}$ represents the fitness value of the worst individual. Generally, a smaller ${Fitness}_{i}$ means that the individual is better at extracting and absorbing the essence of knowledge. Therefore, the individual should have a smaller ${SF}_{i}$, which biases it towards local exploitation. When ${Fitness}_{max}$ is larger, it indicates that the individual is underperforming and needs to bridge the knowledge gap. In this case, the individual should have a larger ${SF}_{i}$ which biases it towards global exploration.

Knowledge acquisition and transformation are inherently lossy processes. For the $k^{th}$ gap vector $\left({Gap}_{k}\right)$,the $i^{th}$ individual absorbs a portion of the knowledge, which is referred to as the $k^{th}$ knowledge gain $\left({KG}_{k}\right)$. For the $i^{th}$ individual, $\left({KG}_{k}\right)$ is obtained by performing the ${LF}_{k}$ and ${SF}_{i}$ operations sequentially on the $k^{th}$group gap vector. This process is described by equation (15):


$$\begin{array}{c} {KG}_{k}={SF}_{i}\times {LF}_{k}\times {Gap}_{k},\;(k=1,2,3,4) \end{array}$$ (15)

Here, ${KG}_{k}$ represents the knowledge gained by the $i^{th}$ individual from the $k^{th}$ gap. ${SF}_{i}$ is the self-evaluation of the individual, while ${LF}_{k}$ is the evaluation of the external situation. Through the combined effect of these two evaluations, the $i^{th}$ individual identifies the knowledge it requires from the ${Gap}_{k}$ thereby completing the learning process. By assimilating the knowledge gaps between different individuals, the $i^{th}$ individual accumulates a wealth of knowledge. The detailed learning process is given by equation (16), which models the improved prey attack phase.


$$\begin{array}{c} x_{i,j}^{new1}={\;x}_{i,j}+\sum_{k=1}^{4}{KG}_{k},\;(k=1,2,3,4) \end{array}$$ (16)

**Fig 3:**
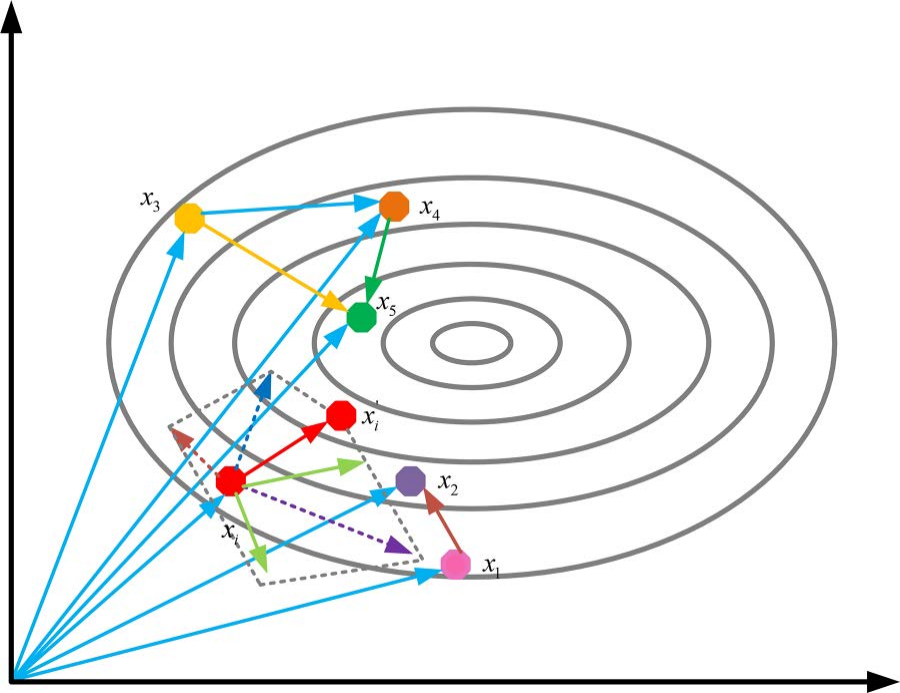
The influence of directional information on the convergence direction.

This mechanism is a collaborative search strategy that employs five vectors, each contributing one of four types of information related to the convergence direction. By balancing these four directional pieces of information based on the distance between vectors and fitness values, the algorithm adaptively determines the current search direction. As shown in Fig 3, the contributions of five individuals generate four search directions, which are interdependent. This effectively reduces the risk of the ASBOA algorithm becoming trapped in local optima.

Therefore, the improved ASBOA exploration phase is updated using the following equation:


$$\begin{array}{c} \begin{array}{c} x_{i\text{,}j}^{new\text{1}}\text{ = }\left\{ \;\;\;\;\;\;\;\;\begin{array}{c} {\text{\textbackslash{} }x}_{i\text{,}j}\text{ + }r_{\text{2}}\times {(x}_{R_{1}}-x_{R_{2}}),\;if\text{\textbackslash{} }iter\text{ < }\frac{\text{1}}{\text{3}}T_{max}\\ x_{best}\text{ + }exp\left({\left(\frac{iter}{T}\right)}^{\text{4}}\right)\times (RB\text{ - 0.5})\times \left(x_{best}\text{ - }x_{i\text{,}j}\right),\;if\text{\textbackslash{} }\frac{\text{1}}{\text{3}}T_{max}\leq iter\text{ < }\frac{\text{2}}{\text{3}}T_{max}\\ {\text{\textbackslash{} \textbackslash{} }x}_{i\text{,}j}+\;\sum_{k\text{ = 1}}^{\text{4}}{KG}_{k},\;(k\text{ = 1,2,3,4})\;,\;else \end{array}\right.\; \end{array} \end{array}$$ (17)

#### 3.2.2 Optimal boundary control mechanism.

During the iterative process of optimization algorithms, some individuals often exceed the predefined search boundaries. In the standard SBOA algorithm, this is typically handled by directly setting individuals that exceed the boundaries to the upper or lower limits of the search space. However, this method fails to effectively utilize the positional information of individuals, potentially resulting in the loss of their original movement trends and valuable information. In the search process of the optimization algorithm, the entire population should focus on exploring new potential positions, with the current global best solution providing critical clues for identifying these new positions. Therefore, to maximize the use of positional information, an optimal boundary control mechanism based on the current global best solution’s position is proposed [60]. By incorporating the global best solution, this mechanism adjusts the position of individuals that exceed the boundaries, as described by equation (18).


$$\begin{array}{c} x_{i,j}(iter+1)=rand\times x_{best},\;x<lb\;\\ x_{i,j}(iter+1)=(1-rand)\times x_{best},\;x>lb \end{array}$$ (18)

**Fig 4:**
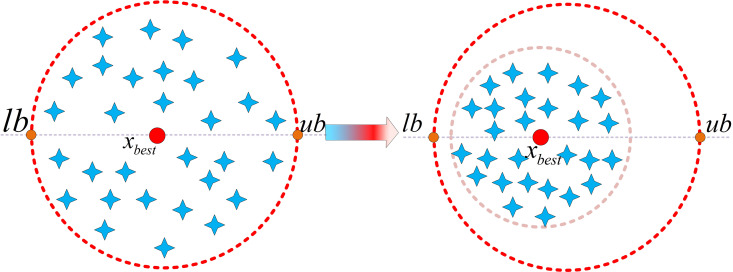
Schematic diagram of optimal boundary control mechanism.

As shown in Fig 4, during the iterative process, the search agents continuously move towards the global best individual, causing the boundaries of the entire search space to shrink towards the optimal individual. This prevents the algorithm from performing ineffective exploration, allowing it to converge more quickly to the vicinity of the optimal solution.

#### 3.2.3 Information retention control mechanism.

Following the adjustment of each individual’s candidate solution during the learning process, its quality may either increase or decrease. Consequently, it is essential to confirm whether actual progress has been achieved. If progress is made, the fitness value ${Fitness}_{i}$ of the $i^{th}$ individual will decrease, resulting in an improvement in its ranking. If the $i^{th}$ individual experiences a regression, the individual may discard some of the knowledge it has acquired. Nonetheless, due to the time and effort involved in the learning process, there remains a small probability that the acquired knowledge may still be preserved [61]. To manage this retention probability, a knowledge retention control mechanism is introduced in ASBOA, with a retention probability (P=0.001). This process is described by equation (19).


$$\begin{array}{c} X_{i}=\left\{ \;\;\;\;\begin{array}{c} \;{\;X}_{i}^{{new}_{m}},\;if\;F_{i}^{{new}_{m}}<F_{i}\\ \left\{ \;\;\;\begin{array}{c} \;\;\;\;{\;X}_{i}^{{new}_{m}},\;if\;r_{5}<P\\ X_{i\;},\;else \end{array}\right.\;,else \end{array}\right.\;\;\;\;\;\;\;,\;(m=1,2) \end{array}$$ (19)

Here, $r_{5}$ is a random number uniformly distributed in the range [0, 1], and PPP is applied to decide whether the newly acquired knowledge of the $i^{th}$ individual should be retained when the individual fails to update. This implies that when an individual fails to update, there is a 0.001 chance that the individual will be carried over to the next generation of the population. Additionally, it ensures that the current global best solution remains unchanged, thereby accelerating the convergence of the algorithm.

In summary, the pseudocode for ASBOA is depicted as **Algorithm 1**.

**Algorithm 1. Pseudo-Code of ASBOA.**

*1: Initialize the secretary bird population $x_{i,j}(i=1,2,\ldots ,N,j=1,2,\ldots ,dim)$ *by Equations (6)**

*2: *Initialize parameter P*$,r_{1},r_{2}\;$* and* l*

*3: Calculate the fitness of each secretary bird*

*4:* ***while*** *t=1:T* ***do***

*5:    [~,ind]=sort(f(x))*

*6:    $x_{best}=x\left(ind(1),:\right)$*

*7:    ****for*** *i=1:N* ***do***

*8.* ***Hunting behavior (exploration):***

**9:*       *Calculate the fitness* $x_{i,j}^{new1}$ *using Equation (17)*.*

*9:       Update the position of the current search agent by Equation (19)*

*10:       Using equation (18) for boundary adjustment*

*11:* ***Escape strategy (exploitation):***

*12:       *Calculate the fitness* $x_{i,j}^{new2}$ *using Equation (6)*.*

*13:       Update the position of the current individual using Equation (19).*

*14:       Using equation (18) for boundary adjustment*

*15:* ***End for***

*16:    Update the best solution found so far $x_{best}$.*

*17:* ***End while***

#### 3.2.4 Computational complexity analysis.

The time required by different algorithms to solve the same optimization problem may vary. Evaluating the computational complexity of an algorithm is a crucial metric for assessing its execution efficiency and practical feasibility [62]. In this study, we conducted a detailed analysis of the time complexity of the SBOA algorithm using Big-O notation. Assuming the population size of the Secretary Bird Optimization Algorithm (SBOA) is N, the problem dimension is dim, and the maximum number of iterations is T, the algorithm’s primary steps can be decomposed and analyzed based on the definition of time complexity and the computation rules of Big-O notation. First, the random initialization of the population, as the initial step of the algorithm, involves generating initial values for N solutions, resulting in a time complexity of O(N). Next, during the solution update process, two main operations are performed: (1) locating the position of the current optimal solution and (2) updating the positions of all solutions. These operations have time complexities of  O(T × N) and O(T × N × dim, respectively. Overall, the total time complexity of the SBOA algorithm can be summarized as O(N × (T × dim + 1)). For the improved algorithm proposed in this study, ASBOA, no additional iterative operations or higher-complexity computations were introduced. Therefore, its time complexity remains consistent with the original SBOA, still O(N × (T × dim + 1)).

#### 3.2.5 Strategy effectiveness analysis.

**Fig 5:**
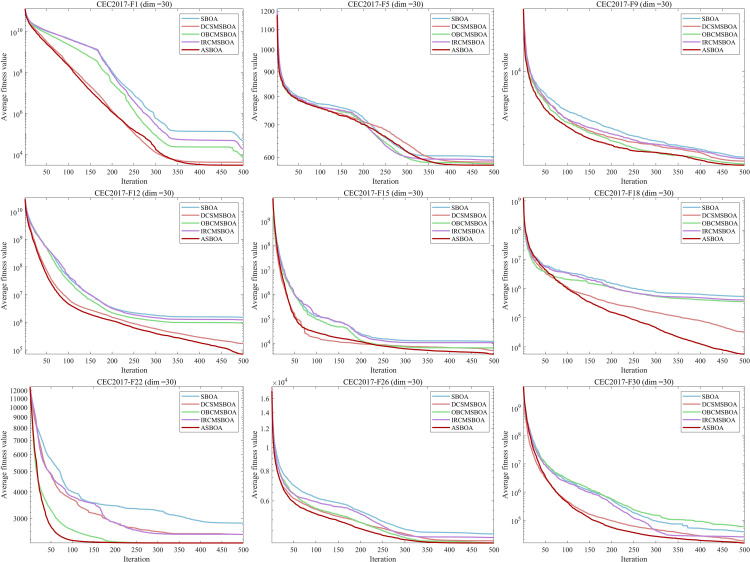
Convergence curves of SBOA improved by different strategies.

This section delves into the impact of three enhancement strategies on the SBOA algorithm: the Differential Cooperative Search Mechanism, Optimal Boundary Control Mechanism, and Information Retention Control Mechanism. Based on these strategies, three variants of SBOA were proposed: DCSMSBOA, OBCMSBOA, and IRCMSBOA. According to the experimental results shown in Fig 5 each strategy significantly improved the convergence accuracy and speed of SBOA, with the ASBOA, which combines all three strategies, demonstrating the best performance. Specifically, for both unimodal and multimodal functions, OBCMSBOA and IRCMSBOA exhibited similar effects, effectively enhancing the convergence speed and accuracy of the algorithm. Among them, the improvement in DCSMSBOA was particularly remarkable, significantly accelerating the convergence speed. Furthermore, when dealing with complex mixed-modal functions, IRCMSBOA showed some decline in performance, whereas OBCMSBOA and DCSMSBOA demonstrated stronger adaptability. Overall, the ASBOA, which integrates all three strategies, achieved excellent performance on most functions.

In conclusion, ASBOA successfully overcame the issues of slow convergence and premature convergence, achieving satisfactory results on multiple benchmark functions, thanks to the effective integration of these three strategies.

## 4. Experimental results and analysis

### 4.1 Test function and compare algorithms parameter Settings

The experiment compares the performance of ASBOA with the standard SBOA, classical algorithms, and recently proposed algorithms on the CEC2017 benchmark suite (dim = 30/100) [12]. Table 1 shows the algorithm information and related parameter settings used in the experiment. The classical algorithms include the Grey Wolf Optimizer (GWO) [44], Whale Optimization Algorithm (WOA) [63], Particle Swarm Optimization (PSO) [29], African vultures optimization algorithm (AVOA) [64]. The recently proposed algorithms include the Crested Porcupine Optimizer (CPO) [65], Black-winged Kite Algorithm (BKA) [66], Dung Beetle Optimizer (DBO) [60] and Secretary Bird Optimization Algorithm(SBOA) [54]. To ensure fairness and eliminate the influence of randomness, all algorithms in this experiment were set with a population size of 30, a maximum of 500 iterations, and each algorithm was run independently 30 times. The results were statistically analyzed for mean (Ave), standard deviation (Std), and average ranking, with the best result for each function highlighted in bold. All experiments were performed in an environment running Windows 11, with a system featuring an Intel(R) Core(TM) i5-13400 processor at 2.5GHz, 16GB of RAM, and MATLAB 2024a software.

**Table 1:** Compare algorithm parameter settings.

Algorithms	Parameter label	Value of the parameter
GWO	a	[0,2]
WOA	r, l, a	[0,1], [-1,1], [0,2]
AVOA	$L_{1},L_{2},w,p_{1},p_{2},p_{3}$	0.8, 0.2, 2.5, 0.6, 0.4, 0.6
PSO	$w_{1},w_{2},c_{1},c_{2}$	0.5, 1.0, 1.5, 1.5
CPO	$\alpha ,\;N_{min},\;T_{f},\;T$	0.1, 80, 0.5, 2
BKA	P, r	0.9, [0,1]
DBO	$P_{percent}$	0.2
SBOA	$CF,l,\;r_{1},r_{2}$	[0,1],{1, 2},[0,1],[0,1]
ASBOA	$P,l,\;r_{1},r_{2}$	0.001,{1, 2},[0,1],[0,1]

### 4.2 Assessing performance with CEC2017 and CEC2022 test suite

This section uses the CEC2017 test suite [12] (with dimensions of 30 and 100) and CEC2022 test suite [67] (with dimensions of 30) to evaluate the effectiveness of ASBOA. Tables 2–4 summarize the test results of 9 comparison algorithms and the ASBOA algorithm on two different dimensions, including the average values and standard deviations of the 30 test functions from the CEC2017 and CEC2022 test suite.

**Table 2:** CEC2017 benchmark function experiment results(dim = 30).

ID	Metric	GWO	WOA	AVOA	PSO	CPO	BKA	DBO	SBOA	ASBOA
F1	Ave	4.6027E + 09	5.8907E + 09	1.6236E + 09	1.4843E + 07	6.8288E + 05	1.1656E + 10	2.9788E + 08	7.7291E + 04	**4.0800E + 03**
	Std	2.3220E + 09	1.9587E + 09	1.1182E + 09	6.3432E + 07	5.0348E + 05	1.1691E + 10	2.3621E + 08	2.5819E + 05	**4.1473E + 03**
F2	Ave	5.9185E + 32	3.6144E + 37	1.7913E + 34	7.5070E + 26	9.9358E + 20	5.5184E + 40	2.3940E + 33	1.6471E + 17	**3.7356E + 15**
	Std	1.9289E + 33	1.7706E + 38	9.8106E + 34	4.0718E + 27	4.3561E + 21	2.4540E + 41	1.2105E + 34	3.8690E + 17	**2.0023E + 16**
F3	Ave	5.6775E + 04	2.5966E + 05	6.5618E + 04	5.7084E + 04	6.7901E + 04	3.6505E + 04	9.6297E + 04	2.6021E + 04	**6.5134E + 03**
	Std	1.0242E + 04	5.0059E + 04	2.4525E + 04	8.1298E + 03	9.8438E + 03	1.9893E + 04	2.3549E + 04	7.2071E + 03	**2.9276E + 03**
F4	Ave	7.3015E + 02	1.3379E + 03	6.7127E + 02	5.3361E + 02	5.2112E + 02	2.3165E + 03	6.6931E + 02	5.1291E + 02	**4.8732E + 02**
	Std	1.4434E + 02	3.0748E + 02	1.9545E + 02	2.9942E + 01	**1.3154E + 01**	3.8850E + 03	1.0363E + 02	2.6558E + 01	2.5438E + 01
F5	Ave	6.5502E + 02	8.5798E + 02	7.1603E + 02	7.3231E + 02	6.9570E + 02	7.5564E + 02	7.7215E + 02	5.8144E + 02	**5.7900E + 02**
	Std	4.8229E + 01	6.5224E + 01	3.4135E + 01	5.1467E + 01	**1.9582E + 01**	4.5777E + 01	5.3247E + 01	2.5674E + 01	2.2622E + 01
F6	Ave	6.1628E + 02	6.8494E + 02	6.2101E + 02	6.5473E + 02	6.0216E + 02	6.5925E + 02	6.4879E + 02	6.0279E + 02	**6.0124E + 02**
	Std	6.3119E + 00	1.2815E + 01	6.6330E + 00	1.0863E + 01	**8.8412E-01**	6.8202E + 00	1.4182E + 01	2.2785E + 00	1.1825E + 00
F7	Ave	9.7091E + 02	1.3175E + 03	1.0043E + 03	1.1730E + 03	9.3810E + 02	1.2087E + 03	1.0194E + 03	8.5492E + 02	**8.2600E + 02**
	Std	5.7900E + 01	8.4473E + 01	3.9250E + 01	7.2950E + 01	**1.9775E + 01**	1.0610E + 02	7.6553E + 01	5.0625E + 01	2.8138E + 01
F8	Ave	9.3588E + 02	1.0620E + 03	1.0041E + 03	9.6257E + 02	9.8414E + 02	9.7984E + 02	1.0567E + 03	**8.7330E + 02**	8.7559E + 02
	Std	4.2072E + 01	4.5227E + 01	2.2604E + 01	3.1411E + 01	**1.3897E + 01**	2.0235E + 01	4.8206E + 01	1.8113E + 01	2.1229E + 01
F9	Ave	3.3910E + 03	1.1396E + 04	2.0797E + 03	5.1832E + 03	1.3860E + 03	5.7756E + 03	6.6170E + 03	1.4837E + 03	**1.3143E + 03**
	Std	1.3963E + 03	3.4884E + 03	9.6650E + 02	9.0619E + 02	**3.9476E + 02**	1.3975E + 03	2.0152E + 03	5.4573E + 02	5.1606E + 02
F10	Ave	6.4496E + 03	7.6397E + 03	7.6290E + 03	5.2927E + 03	7.5963E + 03	5.7485E + 03	6.8589E + 03	4.6612E + 03	**4.0111E + 03**
	Std	1.8843E + 03	6.8272E + 02	7.0935E + 02	7.7019E + 02	**2.8240E + 02**	9.2923E + 02	1.2387E + 03	8.1604E + 02	6.4897E + 02
F11	Ave	2.5793E + 03	9.8359E + 03	1.4613E + 03	1.3065E + 03	1.2688E + 03	2.2345E + 03	1.9285E + 03	1.2228E + 03	**1.1821E + 03**
	Std	1.1361E + 03	3.9854E + 03	6.6039E + 01	7.1243E + 01	**2.7180E + 01**	2.0683E + 03	9.3780E + 02	3.5844E + 01	4.0986E + 01
F12	Ave	2.2996E + 08	4.7786E + 08	9.9594E + 07	2.2575E + 07	1.0652E + 06	6.7461E + 08	6.1397E + 07	1.4114E + 06	**1.3924E + 05**
	Std	2.1206E + 08	2.4226E + 08	1.1931E + 08	2.4596E + 07	6.1056E + 05	1.8826E + 09	7.0768E + 07	1.0752E + 06	**1.5420E + 05**
F13	Ave	5.7815E + 07	1.0057E + 07	4.2441E + 07	1.4128E + 05	2.1747E + 04	1.9257E + 08	5.9153E + 06	2.2753E + 04	**1.6378E + 04**
	Std	8.4087E + 07	5.6213E + 06	1.9267E + 08	8.0237E + 04	**1.1021E + 04**	5.5476E + 08	1.1308E + 07	2.0164E + 04	1.5253E + 04
F14	Ave	4.4813E + 05	2.8355E + 06	1.4853E + 05	8.1058E + 05	2.0656E + 03	1.1240E + 05	2.0776E + 05	2.7835E + 04	**1.6297E + 03**
	Std	3.8252E + 05	2.5348E + 06	1.8101E + 05	8.7494E + 05	5.4490E + 02	5.2811E + 05	1.5650E + 05	2.5862E + 04	**4.9843E + 01**
F15	Ave	4.8656E + 06	5.1188E + 06	1.4229E + 05	4.4987E + 04	4.1610E + 03	1.2197E + 05	2.8109E + 05	1.0821E + 04	**4.1482E + 03**
	Std	1.1945E + 07	4.9724E + 06	1.1749E + 05	2.8039E + 04	**1.5476E + 03**	4.2062E + 05	1.0144E + 06	1.0686E + 04	2.2201E + 03
F16	Ave	2.7882E + 03	4.2061E + 03	3.0306E + 03	3.1570E + 03	3.1372E + 03	3.3655E + 03	3.3962E + 03	2.3734E + 03	**2.3093E + 03**
	Std	4.4420E + 02	5.9123E + 02	3.4510E + 02	4.0488E + 02	**1.9220E + 02**	5.8153E + 02	5.2929E + 02	2.5086E + 02	2.7276E + 02
F17	Ave	2.1575E + 03	2.8210E + 03	2.1427E + 03	2.4937E + 03	2.1018E + 03	2.3156E + 03	2.6303E + 03	1.9690E + 03	**1.9054E + 03**
	Std	2.2730E + 02	3.5496E + 02	1.9831E + 02	3.1663E + 02	1.3373E + 02	2.3682E + 02	2.3263E + 02	1.5691E + 02	**1.2515E + 02**
F18	Ave	4.0070E + 06	8.7364E + 06	2.1940E + 06	3.2171E + 06	1.2279E + 05	1.6525E + 05	3.4820E + 06	5.2582E + 05	**2.6091E + 04**
	Std	7.4913E + 06	7.7302E + 06	2.6563E + 06	3.6747E + 06	1.0084E + 05	1.9090E + 05	4.5648E + 06	3.8420E + 05	**1.6196E + 04**
F19	Ave	2.0097E + 06	2.5522E + 07	3.9645E + 05	1.4499E + 05	7.2330E + 03	8.7509E + 05	3.1456E + 06	9.2431E + 03	**4.3636E + 03**
	Std	3.9236E + 06	2.4387E + 07	7.1115E + 05	1.6106E + 05	5.3160E + 03	3.0888E + 06	6.3774E + 06	1.1073E + 04	**3.1525E + 03**
F20	Ave	2.4916E + 03	2.9419E + 03	2.5075E + 03	2.7330E + 03	2.4922E + 03	2.5692E + 03	2.7565E + 03	**2.2623E + 03**	2.3364E + 03
	Std	2.2614E + 02	2.5868E + 02	1.7410E + 02	1.9168E + 02	1.3857E + 02	1.9947E + 02	1.9604E + 02	**1.2117E + 02**	1.5362E + 02
F21	Ave	2.4314E + 03	2.6485E + 03	2.4961E + 03	2.5327E + 03	2.4824E + 03	2.5636E + 03	2.5611E + 03	2.3665E + 03	**2.3649E + 03**
	Std	3.8452E + 01	5.3092E + 01	2.7385E + 01	5.0534E + 01	**1.4676E + 01**	4.7648E + 01	5.6812E + 01	2.0066E + 01	1.6183E + 01
F22	Ave	5.9670E + 03	8.3915E + 03	5.2684E + 03	6.4821E + 03	2.3107E + 03	5.9380E + 03	5.1184E + 03	2.7074E + 03	**2.3014E + 03**
	Std	2.7141E + 03	1.1989E + 03	3.1449E + 03	2.1880E + 03	3.0080E + 00	1.9834E + 03	2.5364E + 03	1.0576E + 03	**2.3101E + 00**
F23	Ave	2.8157E + 03	3.1449E + 03	2.9503E + 03	2.9801E + 03	2.8433E + 03	3.1064E + 03	2.9837E + 03	**2.7340E + 03**	2.7403E + 03
	Std	5.9871E + 01	1.0612E + 02	9.0010E + 01	8.0152E + 01	**1.6124E + 01**	1.3230E + 02	7.9659E + 01	2.2600E + 01	2.6745E + 01
F24	Ave	2.9901E + 03	3.2905E + 03	3.1149E + 03	3.1336E + 03	3.0194E + 03	3.2893E + 03	3.1666E + 03	2.8968E + 03	**2.8927E + 03**
	Std	6.2950E + 01	8.6366E + 01	7.9857E + 01	1.1126E + 02	**2.2172E + 01**	1.2461E + 02	8.1229E + 01	2.9672E + 01	2.6696E + 01
F25	Ave	3.0381E + 03	3.2481E + 03	2.9788E + 03	2.9463E + 03	2.9113E + 03	3.2521E + 03	2.9751E + 03	2.9166E + 03	**2.8945E + 03**
	Std	4.5243E + 01	7.3595E + 01	3.8086E + 01	3.3171E + 01	1.6496E + 01	4.3111E + 02	6.3021E + 01	1.8459E + 01	**1.5357E + 01**
F26	Ave	5.2368E + 03	8.4632E + 03	5.2479E + 03	6.7757E + 03	5.2332E + 03	7.5591E + 03	7.0555E + 03	4.2742E + 03	**4.2665E + 03**
	Std	**4.1051E + 02**	7.8229E + 02	1.1876E + 03	1.5417E + 03	1.0252E + 03	1.4339E + 03	7.3806E + 02	7.0165E + 02	9.6336E + 02
F27	Ave	3.2747E + 03	3.4649E + 03	3.2718E + 03	3.3083E + 03	3.2764E + 03	3.4012E + 03	3.3430E + 03	**3.2197E + 03**	3.2369E + 03
	Std	3.2270E + 01	1.3181E + 02	4.5448E + 01	5.8450E + 01	1.0555E + 01	1.1055E + 02	7.2699E + 01	**1.0364E + 01**	2.0743E + 01
F28	Ave	3.5110E + 03	3.8754E + 03	3.3900E + 03	3.3203E + 03	3.2697E + 03	4.0330E + 03	3.4302E + 03	3.2564E + 03	**3.2115E + 03**
	Std	1.3557E + 02	2.8679E + 02	8.4041E + 01	3.5672E + 01	2.6502E + 01	1.0626E + 03	1.2173E + 02	2.7772E + 01	**2.3774E + 01**
F29	Ave	3.9982E + 03	5.6369E + 03	4.2045E + 03	4.4217E + 03	4.0253E + 03	4.5253E + 03	4.4956E + 03	**3.5859E + 03**	3.6083E + 03
	Std	2.7911E + 02	7.2516E + 02	2.5532E + 02	3.3079E + 02	**1.3423E + 02**	3.7151E + 02	3.3631E + 02	1.4059E + 02	1.3820E + 02
F30	Ave	1.5848E + 07	6.0262E + 07	3.2593E + 06	1.7860E + 06	1.1947E + 05	9.1625E + 06	3.8208E + 06	4.7996E + 04	**2.8251E + 04**
	Std	1.4117E + 07	4.3714E + 07	2.5084E + 06	1.0305E + 06	7.9113E + 04	2.0586E + 07	4.7949E + 06	8.1250E + 04	**1.9413E + 04**

**Table 3:** CEC2017 benchmark function experiment results(dim = 100).

ID	Metric	GWO	WOA	AVOA	PSO	CPO	BKA	DBO	SBOA	ASBOA
F1	Ave	8.1627E + 10	1.1039E + 11	3.4023E + 10	1.2209E + 10	1.3923E + 10	1.5659E + 11	8.5771E + 10	1.5737E + 10	**2.4025E + 09**
	Std	1.2079E + 10	1.1775E + 10	7.0004E + 09	4.1538E + 09	3.2685E + 09	4.4050E + 10	6.7686E + 10	6.6470E + 09	**2.1810E + 09**
F2	Ave	1.6815E + 143	8.3502E + 175	3.1081E + 128	2.0201E + 143	3.2541E + 127	6.6701E + 167	3.8337E + 154	**1.4262E + 117**	1.4744E + 121
	Std	5.8972E + 143	**6.5535E + 04**	1.6367E + 129	1.0009E + 144	1.7578E + 128	**6.5535E + 04**	**6.5535E + 04**	5.4608E + 117	7.8786E + 121
F3	Ave	5.1650E + 05	9.1532E + 05	5.8899E + 05	4.3642E + 05	4.5532E + 05	2.6826E + 05	6.0173E + 05	3.4653E + 05	**2.5823E + 05**
	Std	7.9944E + 04	1.4066E + 05	1.0843E + 05	1.8923E + 05	4.9811E + 04	3.1512E + 04	2.3957E + 05	4.8887E + 04	**2.4877E + 04**
F4	Ave	9.0876E + 03	2.1697E + 04	3.7694E + 03	2.7978E + 03	2.3811E + 03	3.7308E + 04	1.6354E + 04	1.7709E + 03	**1.1814E + 03**
	Std	2.1705E + 03	4.3326E + 03	1.0212E + 03	8.0101E + 02	4.4831E + 02	2.9648E + 04	1.4657E + 04	4.3475E + 02	**1.5957E + 02**
F5	Ave	1.3686E + 03	1.9425E + 03	1.7065E + 03	1.4034E + 03	1.6516E + 03	1.5678E + 03	1.6552E + 03	1.1748E + 03	**1.0789E + 03**
	Std	1.1045E + 02	1.0522E + 02	7.6141E + 01	4.7614E + 01	**4.4734E + 01**	1.6934E + 02	2.0786E + 02	6.4626E + 01	7.0628E + 01
F6	Ave	6.5501E + 02	7.0729E + 02	6.7237E + 02	6.6786E + 02	6.4105E + 02	6.8126E + 02	6.7985E + 02	6.3850E + 02	**6.3473E + 02**
	Std	4.4009E + 00	9.6743E + 00	1.1530E + 01	**3.6854E + 00**	5.9084E + 00	8.3803E + 00	1.3186E + 01	5.2871E + 00	7.2246E + 00
F7	Ave	2.4992E + 03	3.7358E + 03	2.4938E + 03	3.2361E + 03	2.3836E + 03	3.3919E + 03	2.9524E + 03	2.3944E + 03	**2.1076E + 03**
	Std	1.1968E + 02	1.4941E + 02	1.6323E + 02	1.3862E + 02	**1.1288E + 02**	1.5205E + 02	3.0192E + 02	1.7692E + 02	1.7007E + 02
F8	Ave	1.6957E + 03	2.3757E + 03	2.0114E + 03	1.8110E + 03	1.9829E + 03	2.0504E + 03	2.1674E + 03	1.5413E + 03	**1.3923E + 03**
	Std	1.0785E + 02	9.4640E + 01	8.4019E + 01	7.3014E + 01	**6.2774E + 01**	1.6873E + 02	2.3966E + 02	7.7929E + 01	8.9841E + 01
F9	Ave	5.1107E + 04	7.9810E + 04	6.1771E + 04	2.9942E + 04	4.7973E + 04	3.8158E + 04	7.5716E + 04	2.7180E + 04	**2.5191E + 04**
	Std	9.2381E + 03	1.3387E + 04	1.7620E + 04	**2.7638E + 03**	6.9991E + 03	1.1640E + 04	9.5710E + 03	5.1791E + 03	4.8123E + 03
F10	Ave	2.6349E + 04	2.9108E + 04	3.0041E + 04	1.8525E + 04	3.0804E + 04	2.0476E + 04	2.7164E + 04	1.6969E + 04	**1.6685E + 04**
	Std	6.3356E + 03	1.6179E + 03	1.3253E + 03	2.3244E + 03	**5.3352E + 02**	3.8242E + 03	4.5203E + 03	1.4771E + 03	1.4892E + 03
F11	Ave	8.7030E + 04	2.6835E + 05	8.6258E + 04	1.0611E + 05	9.4869E + 04	7.3728E + 04	2.3424E + 05	3.5870E + 04	**1.8439E + 04**
	Std	1.6759E + 04	8.3832E + 04	3.1259E + 04	2.2486E + 04	1.5332E + 04	5.3150E + 04	5.3418E + 04	1.2132E + 04	**5.1933E + 03**
F12	Ave	2.0156E + 10	3.0355E + 10	1.5464E + 10	9.2784E + 08	8.3200E + 08	6.1021E + 10	7.4815E + 09	3.2185E + 08	**8.5244E + 07**
	Std	7.9450E + 09	5.3706E + 09	8.8599E + 09	3.7592E + 08	1.9934E + 08	4.1211E + 10	2.4971E + 09	1.1544E + 08	**3.8464E + 07**
F13	Ave	3.2778E + 09	2.7431E + 09	1.1729E + 09	8.8627E + 05	1.7755E + 05	1.0706E + 10	2.8879E + 08	3.9689E + 05	**1.1022E + 04**
	Std	1.6700E + 09	9.6783E + 08	1.2953E + 09	2.7719E + 06	1.0759E + 05	9.9726E + 09	2.2658E + 08	1.7547E + 06	**4.3376E + 03**
F14	Ave	8.7662E + 06	2.3398E + 07	1.2231E + 07	9.4961E + 06	4.2768E + 06	3.2616E + 06	1.6195E + 07	3.6101E + 06	**5.0188E + 05**
	Std	4.4618E + 06	8.4092E + 06	5.0261E + 06	5.3613E + 06	1.9950E + 06	3.8403E + 06	1.0292E + 07	2.1867E + 06	**2.8425E + 05**
F15	Ave	4.5248E + 08	4.4400E + 08	4.5995E + 08	1.6129E + 05	9.8113E + 03	2.8410E + 09	6.6173E + 07	1.4506E + 04	**4.8196E + 03**
	Std	5.7468E + 08	2.0600E + 08	5.9208E + 08	3.1141E + 05	**3.0233E + 03**	5.2798E + 09	1.1363E + 08	3.3853E + 04	3.9282E + 03
F16	Ave	7.5534E + 03	1.6874E + 04	9.8400E + 03	7.7752E + 03	1.0315E + 04	1.0863E + 04	9.4588E + 03	5.4975E + 03	**5.2308E + 03**
	Std	1.2377E + 03	2.7276E + 03	6.0531E + 02	7.7170E + 02	5.6558E + 02	4.1039E + 03	1.9656E + 03	6.4089E + 02	**5.6236E + 02**
F17	Ave	6.3390E + 03	2.6347E + 04	8.0398E + 03	6.6399E + 03	6.9283E + 03	3.7569E + 04	9.0508E + 03	5.0501E + 03	**4.3839E + 03**
	Std	1.6197E + 03	2.5914E + 04	7.9129E + 02	7.0075E + 02	**4.0460E + 02**	8.0983E + 04	1.2045E + 03	6.3883E + 02	5.0968E + 02
F18	Ave	1.2730E + 07	1.5355E + 07	1.8729E + 07	7.8847E + 06	5.3850E + 06	1.6856E + 07	2.8409E + 07	4.7219E + 06	**9.3643E + 05**
	Std	1.0567E + 07	7.9635E + 06	8.4628E + 06	3.6961E + 06	2.2523E + 06	4.4932E + 07	1.5742E + 07	2.2046E + 06	**5.1013E + 05**
F19	Ave	6.4167E + 08	5.1854E + 08	2.9899E + 08	3.0467E + 06	1.2608E + 04	2.5379E + 09	8.5241E + 07	8.2436E + 03	**5.9797E + 03**
	Std	6.0669E + 08	3.2211E + 08	2.4101E + 08	2.7465E + 06	1.0569E + 04	4.9608E + 09	5.9769E + 07	6.3005E + 03	**5.1475E + 03**
F20	Ave	6.3628E + 03	7.1164E + 03	7.0111E + 03	5.9706E + 03	7.2599E + 03	5.6554E + 03	7.3577E + 03	4.9848E + 03	**4.8395E + 03**
	Std	1.1464E + 03	4.8065E + 02	4.8934E + 02	5.1726E + 02	**3.1480E + 02**	4.3774E + 02	7.2746E + 02	5.1440E + 02	5.4989E + 02
F21	Ave	3.2639E + 03	4.4929E + 03	3.7311E + 03	3.7390E + 03	3.3792E + 03	4.1228E + 03	4.0309E + 03	2.9512E + 03	**2.8436E + 03**
	Std	1.6152E + 02	1.8417E + 02	1.1092E + 02	2.1890E + 02	**5.3332E + 01**	2.8966E + 02	1.7079E + 02	6.8074E + 01	9.5788E + 01
F22	Ave	2.9855E + 04	3.1729E + 04	3.2753E + 04	2.1763E + 04	3.2973E + 04	2.3821E + 04	2.9222E + 04	**1.9092E + 04**	1.9555E + 04
	Std	6.1213E + 03	1.7661E + 03	1.3897E + 03	1.8213E + 03	**9.9487E + 02**	3.2508E + 03	4.6128E + 03	4.9149E + 03	3.4403E + 03
F23	Ave	3.8574E + 03	5.3473E + 03	4.8203E + 03	4.3475E + 03	3.9937E + 03	5.1303E + 03	4.7240E + 03	**3.3962E + 03**	3.4285E + 03
	Std	9.5350E + 01	2.5995E + 02	3.5392E + 02	1.8445E + 02	**5.2134E + 01**	4.1083E + 02	2.9378E + 02	8.1769E + 01	9.6113E + 01
F24	Ave	4.8355E + 03	6.7354E + 03	5.9567E + 03	5.4747E + 03	4.5634E + 03	6.9374E + 03	6.2052E + 03	**4.0239E + 03**	4.0752E + 03
	Std	2.0191E + 02	4.3351E + 02	5.1883E + 02	3.8061E + 02	**6.5924E + 01**	7.1080E + 02	4.5925E + 02	8.3326E + 01	1.2777E + 02
F25	Ave	8.4239E + 03	1.1002E + 04	5.5970E + 03	4.7997E + 03	4.9310E + 03	1.3234E + 04	1.1432E + 04	4.2326E + 03	**3.8746E + 03**
	Std	9.6393E + 02	1.0215E + 03	7.5256E + 02	2.9156E + 02	3.5402E + 02	4.5584E + 03	7.0276E + 03	2.2783E + 02	**1.4000E + 02**
F26	Ave	1.9796E + 04	3.8174E + 04	1.9808E + 04	2.6113E + 04	2.1053E + 04	3.7476E + 04	2.6100E + 04	1.8669E + 04	**1.8074E + 04**
	Std	1.7222E + 03	4.0382E + 03	3.4478E + 03	3.2695E + 03	**1.4967E + 03**	7.7495E + 03	3.0533E + 03	3.6526E + 03	4.2606E + 03
F27	Ave	4.5998E + 03	6.2748E + 03	3.9520E + 03	4.4476E + 03	4.2060E + 03	6.2062E + 03	4.6967E + 03	**3.7336E + 03**	3.8995E + 03
	Std	2.1567E + 02	7.2518E + 02	2.1677E + 02	3.5944E + 02	1.0177E + 02	1.2058E + 03	5.3100E + 02	**9.6446E + 01**	1.5730E + 02
F28	Ave	1.1230E + 04	1.5162E + 04	7.0611E + 03	5.8621E + 03	6.1154E + 03	1.7376E + 04	1.8782E + 04	5.0339E + 03	**4.3588E + 03**
	Std	1.3497E + 03	1.1805E + 03	2.5800E + 03	6.6161E + 02	5.8837E + 02	3.9599E + 03	5.2733E + 03	4.1148E + 02	**3.5816E + 02**
F29	Ave	1.0546E + 04	2.0622E + 04	1.1204E + 04	9.9529E + 03	1.0308E + 04	1.7491E + 04	1.2484E + 04	7.2246E + 03	**7.1907E + 03**
	Std	7.7965E + 02	3.5852E + 03	6.5963E + 02	1.2442E + 03	**4.0208E + 02**	2.3441E + 04	2.8336E + 03	7.2325E + 02	6.0593E + 02
F30	Ave	2.5203E + 09	2.7949E + 09	8.8451E + 08	6.8942E + 07	6.5988E + 06	4.4661E + 09	2.4111E + 08	1.0543E + 06	**4.9827E + 05**
	Std	1.7069E + 09	1.1778E + 09	5.8621E + 08	3.0832E + 07	4.0942E + 06	4.5371E + 09	1.2250E + 08	9.4415E + 05	**4.2619E + 05**

**Table 4:** CEC2022 benchmark function experiment results(dim = 20).

ID	Metric	GWO	WOA	AVOA	PSO	CPO	BKA	DBO	SBOA	ASBOA
F1	Ave	1.5045E + 04	3.9432E + 04	5.9647E + 03	2.1850E + 04	1.2590E + 04	6.6160E + 03	3.8336E + 04	2.6367E + 03	**3.4377E + 02**
	Std	3.6442E + 03	1.1962E + 04	2.4314E + 03	8.6053E + 03	2.9691E + 03	7.9100E + 03	1.3227E + 04	1.7940E + 03	**4.1912E + 01**
F2	Ave	5.1986E + 02	6.1166E + 02	4.7644E + 02	4.9437E + 02	4.6254E + 02	5.8181E + 02	4.9167E + 02	4.6640E + 02	**4.5017E + 02**
	Std	5.4982E + 01	7.9513E + 01	2.5992E + 01	3.8936E + 01	**1.1309E + 01**	2.2048E + 02	5.0699E + 01	2.3655E + 01	1.6099E + 01
F3	Ave	6.0873E + 02	6.7325E + 02	6.1131E + 02	6.4703E + 02	6.0029E + 02	6.5165E + 02	6.3787E + 02	6.0068E + 02	**6.0019E + 02**
	Std	3.1977E + 00	1.4247E + 01	4.8708E + 00	8.2229E + 00	**1.7604E-01**	8.9350E + 00	1.5282E + 01	2.2734E + 00	4.5843E-01
F4	Ave	8.8671E + 02	9.3580E + 02	9.0707E + 02	8.9007E + 02	9.0241E + 02	8.8305E + 02	9.1260E + 02	**8.3893E + 02**	8.4484E + 02
	Std	3.8216E + 01	2.7288E + 01	1.7241E + 01	1.9008E + 01	**1.1082E + 01**	2.2837E + 01	2.8315E + 01	1.2066E + 01	1.5469E + 01
F5	Ave	1.4008E + 03	4.1011E + 03	1.0477E + 03	2.3986E + 03	**9.1471E + 02**	2.0894E + 03	2.4622E + 03	9.6676E + 02	9.4725E + 02
	Std	3.7638E + 02	1.4620E + 03	1.1872E + 02	3.3868E + 02	**2.1238E + 01**	2.9499E + 02	7.1256E + 02	1.0817E + 02	1.4281E + 02
F6	Ave	1.0394E + 07	6.0639E + 06	2.6451E + 06	5.4096E + 03	1.8402E + 04	4.7515E + 06	9.1529E + 05	6.4370E + 03	**4.2304E + 03**
	Std	1.8239E + 07	7.3043E + 06	4.9267E + 06	4.3230E + 03	8.5434E + 03	2.1338E + 07	1.6243E + 06	6.0192E + 03	**2.7792E + 03**
F7	Ave	2.0959E + 03	2.2426E + 03	2.1097E + 03	2.1770E + 03	2.0650E + 03	2.1268E + 03	2.1344E + 03	**2.0429E + 03**	2.0429E + 03
	Std	3.8107E + 01	7.1472E + 01	4.3330E + 01	5.8662E + 01	1.2304E + 01	4.1327E + 01	4.4251E + 01	**1.1659E + 01**	1.4823E + 01
F8	Ave	2.2357E + 03	2.2974E + 03	2.2923E + 03	2.2585E + 03	2.2323E + 03	2.2867E + 03	2.3316E + 03	**2.2275E + 03**	2.2297E + 03
	Std	1.2923E + 01	8.4007E + 01	6.9865E + 01	4.4529E + 01	**1.4389E + 00**	6.6618E + 01	8.9683E + 01	4.4516E + 00	2.2361E + 01
F9	Ave	2.5147E + 03	2.5837E + 03	2.4982E + 03	2.4887E + 03	2.4817E + 03	2.5240E + 03	2.5076E + 03	2.4808E + 03	**2.4808E + 03**
	Std	1.9040E + 01	3.9644E + 01	2.7473E + 01	7.1436E + 00	4.1531E-01	5.6014E + 01	2.7049E + 01	3.3958E-03	**6.4292E-04**
F10	Ave	3.6407E + 03	5.0413E + 03	4.1329E + 03	3.8400E + 03	**2.5270E + 03**	4.4677E + 03	3.2652E + 03	2.7596E + 03	2.7005E + 03
	Std	1.4713E + 03	1.1347E + 03	9.7405E + 02	9.0026E + 02	**6.7741E + 01**	1.1469E + 03	1.1733E + 03	4.3208E + 02	2.9444E + 02
F11	Ave	3.7446E + 03	4.3326E + 03	3.4297E + 03	2.9639E + 03	**2.8889E + 03**	4.6588E + 03	3.3185E + 03	2.9068E + 03	2.9400E + 03
	Std	2.9497E + 02	1.2394E + 03	3.8123E + 02	1.6866E + 02	1.0494E + 02	1.5833E + 03	8.2955E + 02	9.4485E + 01	**4.9827E + 01**
F12	Ave	2.9735E + 03	3.1214E + 03	3.0028E + 03	3.0020E + 03	2.9880E + 03	3.0758E + 03	3.0624E + 03	**2.9435E + 03**	2.9668E + 03
	Std	1.7546E + 01	1.3561E + 02	4.9648E + 01	3.9451E + 01	1.1415E + 01	1.1574E + 02	7.9996E + 01	**6.4081E + 00**	1.4809E + 01

For the unimodal functions CEC2017 F1–F3 and CEC2022 F1-F5, ASBOA demonstrates the ability to converge to a solution close to the global optimum for both the 30-dimensional and 100-dimensional versions of the F1 function. SBOA and CPO only converge to the global optimum in the 30-dimensional case, while other comparison algorithms fail to find better solutions. The CEC2017-F2 function induces significant instability in most algorithms as the dimensionality increases. However, ASBOA effectively addresses this issue, showing notably superior performance. For the CEC2017-F3 function, the global optimum is located within a large, smooth region, which leads to a rapid decline in convergence speed for most algorithms. In contrast, ASBOA excels by avoiding incorrect convergence directions and enhancing convergence speed. Furthermore, while the performance of all algorithms deteriorates with higher dimensions, ASBOA is less affected by dimensionality increases. This is due to the three enhancement mechanisms within ASBOA, which enable the algorithm to better adapt to the search space and deliver improved results.

As the problem dimension grows, the performance of all algorithms in identifying the optimal solution diminishes for the simple multimodal functions CEC2017 F4-F10 and CEC2022 F6-F8. Both ASBOA and SBOA demonstrate superior optimization performance in lower dimensions. However, ASBOA manages to maintain consistent stability and delivers high-quality results across various dimensions. On the other hand, the performance of the standard SBOA gradually worsens as the dimension increases, highlighting the increasing advantages of ASBOA.

For the hybrid functions CEC2017 F11-F20 and CEC2022 F9-F12, ASBOA outperforms the other algorithms in terms of results. In particular, ASBOA maintains its dominance on functions CEC2017-F12, F15, and F18. SBOA also has some advantages, particularly on functions CEC2017-F16, F17, and F20. However, for the majority of the functions, ASBOA’s superiority is the most pronounced.

For the composite functions CEC2017 F21–F30, ASBOA still demonstrates dominant performance on these problems. SBOA and CPO only show advantages in a few cases, while other comparison algorithms exhibit no significant advantage and fail to solve these problems effectively in both dimensions. In summary, the three improvement mechanisms of ASBOA effectively iterated the solutions, leading to competitive performance.

**Fig 6:**
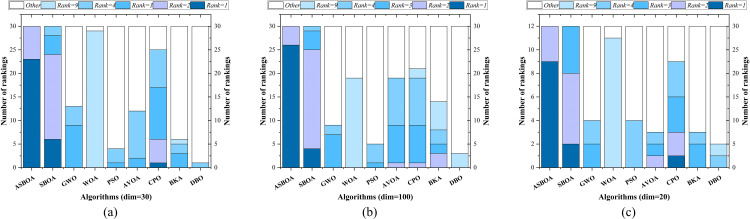
Ranking distribution statistics.

To better illustrate the performance of each algorithm, the ranking distribution of each algorithm across all functions is shown in Fig 6. It is evident that for CEC2017 (dim = 30), ASBOA ranks best in 23 functions and second in 7 functions. For CEC2017 (dim = 100), ASBOA achieves the best performance in 26 functions and ranks second in 4 functions. For the CEC2022 benchmark with dimension = 20, ASBOA achieved first place rankings on 9 out of 12 test functions and secured second place on 2 functions, demonstrating consistently excellent performance without any worst-case rankings. From the ranking perspective, it is noteworthy that ASBOA consistently maintains a top two position across all 42 functions, demonstrating its stability in performance. Notably, ASBOA excels in solving unimodal and composite functions, while the standard SBOA achieves the best result in only 6 functions (dim = 30) and 4 functions (dim = 100). CPO achieves only 1 best result in dim = 30, with other algorithms failing to achieve the best result in any function. This highlights the significant improvement of ASBOA over the standard SBOA.

### 4.3 Convergence analysis

**Fig 7:**
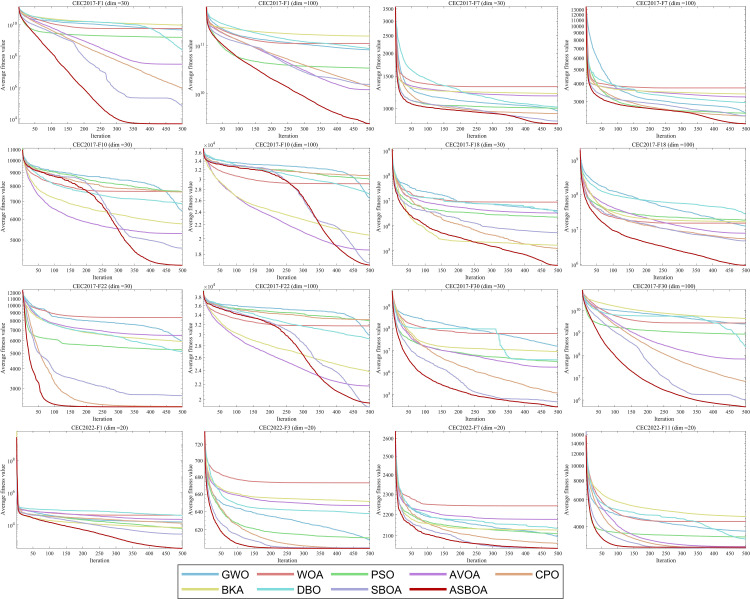
Convergence curves of different algorithms on CEC2017 functions (dim = 30,100) and CEC2022(dim = 30).

To explore the convergence patterns of various algorithms throughout the iteration process and their responsiveness to different functions and dimensions, this study conducted experiments using some representative functions from the CEC 2017 and CEC2022 benchmark suite. These functions include the unimodal function CEC2017-F1 and CEC2022 F1-F3, multimodal functions CEC2017-(F7, and F10) and CEC2022-F7, hybrid functions CEC2017-F18 and CEC2022-F11, as well as composite functions CEC2017-(F22 and F30). The experiments were carried out at dimensions of dim = 30 and dim = 100. The comprehensive convergence results are shown in Fig 7.

For the unimodal function CEC2017-F1 and CEC2022-F1 and F2 convergence curve, ASBOA appears to have an advantage in both convergence speed and accuracy. ASBOA notably reduces the sensitivity of SBOA to parameter variations. Furthermore, the performance advantage of ASBOA becomes especially pronounced in the case of F2, particularly as the problem dimension grows.

For the convergence curves of the multimodal functions CEC2017-(F7 and F1) and CEC2022-F7, ASBOA exhibits the fastest convergence rate and the highest accuracy in reaching the optimal solution. In contrast, other algorithms tend to get trapped in local optima as the number of iterations increases due to the local optimum characteristics of the problem, preventing them from obtaining better solutions. Furthermore, as the dimension increases, ASBOA’s convergence curve stands out among all the algorithms. This demonstrates the effectiveness and competitiveness of the three strategies integrated into ASBOA.

For the hybrid functions CEC2017-F18 and CEC2022-F11 convergence curves, ASBOA also provides the best results. Despite the complex topology of these functions, ASBOA’s convergence curve continues to decrease until the end of the iterations, indicating that it consistently improves the quality of the current solution. This demonstrates ASBOA’s ability to effectively navigate through challenging optimization landscapes.

For the combinatorial functions CEC2017 F22 and F30 convergence curves, ASBOA does not show a significant advantage on the high-dimensional F22, but it still performs relatively well compared to other algorithms. On F30, the results of SBOA and ASBOA are similar, but ASBOA’s relative advantage is more noticeable. In conclusion, it can be deduced that ASBOA exhibits good convergence behavior on these problems, particularly in terms of maintaining stability and finding competitive solutions.

### 4.5 Robustness analysis

**Fig 8:**
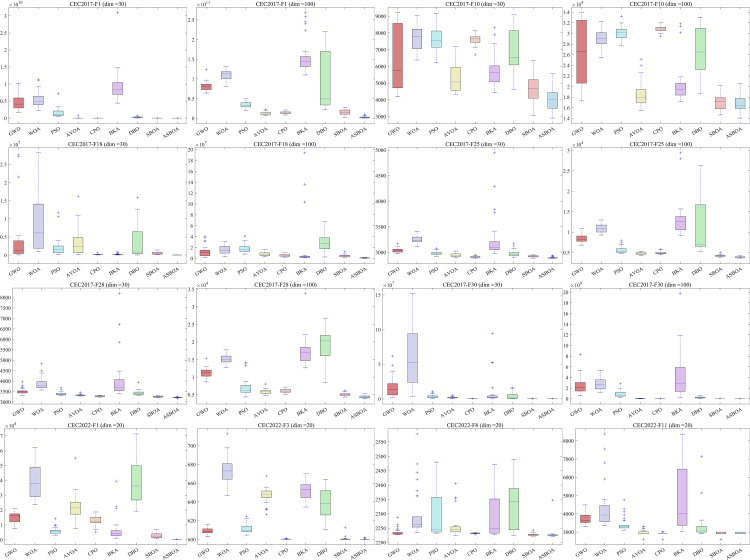
Box plots of different algorithms on CEC2017 functions (dim = 30,100) and CEC2022 functions (dim = 20).

To further confirm the robustness and stability of the algorithm, boxplots illustrating the performance of each algorithm on representative functions were generated, as shown in Fig 8. The figure clearly reveals that the data distribution for ASBOA is generally more concentrated, indicating its robust and stable search capability. This can be attributed to the three enhancement mechanisms embedded within ASBOA, which continuously refine the search process and mitigate the impact of complex problems. Additionally, while the data distributions for GWO, PSO, CPO, and SBOA are relatively stable, their boxplots are positioned higher, indirectly suggesting that these algorithms may not offer a distinct advantage, with their robustness and stability falling short compared to ASBOA.

### 4.6 Wilcoxon rank sum test

To comprehensively demonstrate the superiority of the proposed algorithm, this section uses the Wilcoxon rank-sum test to evaluate the performance of ASBOA in each experiment and compares it with other algorithms at a significance level of p = 0.05. The null hypothesis $\left(H_{0}\right)$ is that there is no significant difference between the two algorithms. When the p-value is less than 0.05, the null hypothesis is rejected, indicating a significant difference between the two algorithms. When the p-value is greater than 0.05, the null hypothesis is accepted, suggesting that the performance difference between the two algorithms is not significant, i.e., the algorithms perform similarly. The differences between the algorithms are presented in graphical form, with sections where the p-value is greater than 0.05 highlighted. The test results are shown in Tables 5–7.

**Table 5:** P-value on CEC2017 (dim = 30).

Function	GWO	WOA	AVOA	PSO	CPO	BKA	DBO	SBOA
F1	3.0199E-11	3.0199E-11	3.0199E-11	6.6955E-11	3.0199E-11	3.0199E-11	3.0199E-11	4.5726E-09
F2	3.0199E-11	3.0199E-11	3.0199E-11	3.3384E-11	4.0772E-11	3.0199E-11	3.0199E-11	1.7294E-07
F3	3.0199E-11	3.0199E-11	3.0199E-11	3.0199E-11	3.0199E-11	3.3384E-11	3.0199E-11	3.6897E-11
F4	3.0199E-11	3.0199E-11	3.0199E-11	2.1947E-08	2.8314E-08	3.0199E-11	3.0199E-11	2.2780E-05
F5	1.4110E-09	3.0199E-11	3.0199E-11	3.0199E-11	3.0199E-11	3.0199E-11	3.0199E-11	**6.3088E-01**
F6	3.0199E-11	3.0199E-11	3.0199E-11	3.0199E-11	3.5638E-04	3.0199E-11	3.0199E-11	4.7138E-04
F7	3.6897E-11	3.0199E-11	3.0199E-11	3.0199E-11	3.0199E-11	3.0199E-11	3.0199E-11	3.7782E-02
F8	1.4294E-08	3.0199E-11	3.0199E-11	4.5043E-11	3.0199E-11	4.0772E-11	3.0199E-11	**1.0000E + 00**
F9	8.8910E-10	3.0199E-11	1.5292E-05	3.0199E-11	**1.1882E-01**	3.6897E-11	3.0199E-11	**1.1536E-01**
F10	3.6459E-08	3.0199E-11	3.0199E-11	7.0881E-08	3.0199E-11	6.7220E-10	8.9934E-11	2.1566E-03
F11	3.0199E-11	3.0199E-11	3.0199E-11	3.4971E-09	3.1967E-09	4.5043E-11	3.6897E-11	4.3531E-05
F12	3.0199E-11	3.0199E-11	3.0199E-11	3.0199E-11	8.1014E-10	3.0199E-11	3.0199E-11	3.1589E-10
F13	3.0199E-11	3.0199E-11	3.0199E-11	1.6132E-10	2.2360E-02	3.6897E-11	8.8910E-10	**3.4783E-01**
F14	3.0199E-11	3.0199E-11	3.0199E-11	3.0199E-11	5.9673E-09	3.0199E-11	3.0199E-11	3.0199E-11
F15	3.0199E-11	3.0199E-11	3.0199E-11	3.0199E-11	**4.6427E-01**	6.6955E-11	1.9568E-10	1.0763E-02
F16	1.1674E-05	3.0199E-11	1.0702E-09	1.9568E-10	3.0199E-11	5.4941E-11	1.7769E-10	7.6183E-03
F17	6.2828E-06	6.0658E-11	6.7362E-06	2.0338E-09	4.1178E-06	3.8249E-09	3.3384E-11	**1.2235E-01**
F18	6.0658E-11	3.0199E-11	3.0199E-11	3.0199E-11	1.6132E-10	3.1589E-10	6.0658E-11	3.0199E-11
F19	3.0199E-11	3.0199E-11	3.0199E-11	1.0937E-10	1.3272E-02	5.4941E-11	3.3384E-11	3.0317E-02
F20	7.9590E-03	8.9934E-11	7.2951E-04	6.5183E-09	3.3679E-04	2.5974E-05	1.4110E-09	**9.0490E-02**
F21	7.3891E-11	3.0199E-11	3.0199E-11	3.0199E-11	3.0199E-11	3.0199E-11	3.0199E-11	9.9410E-04
F22	3.0199E-11	3.0199E-11	3.0199E-11	1.7769E-10	4.9752E-11	3.0199E-11	3.0199E-11	2.7726E-05
F23	1.8500E-08	3.0199E-11	3.0199E-11	3.0199E-11	3.0199E-11	3.0199E-11	3.0199E-11	**3.6322E-01**
F24	1.6947E-09	3.0199E-11	3.0199E-11	3.0199E-11	3.0199E-11	3.0199E-11	3.0199E-11	7.0617E-03
F25	3.0199E-11	3.0199E-11	1.0937E-10	1.6947E-09	1.0188E-05	3.0199E-11	3.4742E-10	3.3681E-05
F26	5.1857E-07	3.0199E-11	2.6243E-03	2.8314E-08	4.4205E-06	6.7220E-10	6.0658E-11	**8.3026E-01**
F27	1.6062E-06	3.6897E-11	9.2113E-05	3.1967E-09	1.3111E-08	6.0658E-11	2.0338E-09	1.1058E-04
F28	3.0199E-11	3.0199E-11	3.0199E-11	3.0199E-11	1.8567E-09	3.0199E-11	3.0199E-11	8.3520E-08
F29	7.0881E-08	3.0199E-11	1.6132E-10	4.9752E-11	3.1589E-10	4.5043E-11	3.0199E-11	**3.9527E-01**
F30	3.0199E-11	3.0199E-11	3.0199E-11	3.0199E-11	2.6695E-09	3.0199E-11	7.1186E-09	7.3940E-03

**Table 6:** P-value on CEC2017 (dim = 100).

Function	GWO	WOA	PSO	AVOA	CPO	BKA	DBO	SBOA
F1	3.0199E-11	3.0199E-11	3.0199E-11	8.1527E-11	3.6897E-11	3.0199E-11	3.0199E-11	1.7769E-10
F2	6.6955E-11	3.0199E-11	2.6099E-10	3.0199E-11	2.3168E-06	3.0199E-11	3.0199E-11	1.0315E-02
F3	3.0199E-11	3.0199E-11	3.0199E-11	3.0199E-11	3.0199E-11	**4.9178E-01**	3.0199E-11	9.9186E-11
F4	3.0199E-11	3.0199E-11	3.0199E-11	3.0199E-11	3.0199E-11	3.0199E-11	3.0199E-11	1.2870E-09
F5	3.0199E-11	3.0199E-11	3.0199E-11	3.0199E-11	3.0199E-11	3.0199E-11	3.0199E-11	6.7362E-06
F6	6.0658E-11	3.0199E-11	3.0199E-11	3.0199E-11	1.2362E-03	3.0199E-11	3.0199E-11	3.9167E-02
F7	3.1589E-10	3.0199E-11	8.8910E-10	3.0199E-11	4.3106E-08	3.0199E-11	3.0199E-11	3.0103E-07
F8	1.0937E-10	3.0199E-11	3.0199E-11	3.0199E-11	3.0199E-11	3.0199E-11	3.0199E-11	2.0283E-07
F9	4.9752E-11	3.0199E-11	4.0772E-11	2.6806E-04	4.0772E-11	9.7555E-10	3.0199E-11	**1.4945E-01**
F10	2.6099E-10	3.0199E-11	3.0199E-11	6.2027E-04	3.0199E-11	5.5329E-08	4.5043E-11	**5.1060E-01**
F11	3.0199E-11	3.0199E-11	3.0199E-11	3.0199E-11	3.0199E-11	3.0199E-11	3.0199E-11	9.8329E-08
F12	3.0199E-11	3.0199E-11	3.0199E-11	3.3384E-11	3.0199E-11	3.0199E-11	3.0199E-11	5.4941E-11
F13	3.0199E-11	3.0199E-11	3.0199E-11	3.0199E-11	3.0199E-11	3.0199E-11	3.0199E-11	7.3891E-11
F14	3.0199E-11	3.0199E-11	3.0199E-11	4.9752E-11	3.0199E-11	6.7220E-10	3.0199E-11	4.9752E-11
F15	3.0199E-11	3.0199E-11	3.0199E-11	3.0199E-11	1.5964E-07	3.0199E-11	3.0199E-11	7.6588E-05
F16	4.5043E-11	3.0199E-11	3.0199E-11	3.0199E-11	3.0199E-11	3.0199E-11	3.0199E-11	**2.0095E-01**
F17	1.6132E-10	3.0199E-11	3.0199E-11	4.5043E-11	3.0199E-11	4.0772E-11	3.0199E-11	4.9426E-05
F18	3.6897E-11	3.0199E-11	3.0199E-11	3.0199E-11	3.0199E-11	4.9980E-09	3.0199E-11	4.0772E-11
F19	3.0199E-11	3.0199E-11	3.0199E-11	3.0199E-11	7.2208E-06	3.0199E-11	3.0199E-11	2.4157E-02
F20	2.1959E-07	3.3384E-11	4.0772E-11	1.8500E-08	3.0199E-11	1.5964E-07	8.9934E-11	**3.4783E-01**
F21	3.3384E-11	3.0199E-11	3.0199E-11	3.0199E-11	3.0199E-11	3.0199E-11	3.0199E-11	2.9590E-05
F22	6.7220E-10	3.0199E-11	3.0199E-11	6.9125E-04	3.0199E-11	1.5465E-09	9.9186E-11	**6.9522E-01**
F23	3.0199E-11	3.0199E-11	3.0199E-11	3.0199E-11	3.0199E-11	3.0199E-11	3.0199E-11	**2.4581E-01**
F24	3.0199E-11	3.0199E-11	3.0199E-11	3.0199E-11	3.0199E-11	3.0199E-11	3.0199E-11	**1.8577E-01**
F25	3.0199E-11	3.0199E-11	3.0199E-11	3.0199E-11	3.0199E-11	3.0199E-11	3.0199E-11	4.9980E-09
F26	**5.7460E-02**	3.0199E-11	3.3874E-02	4.5726E-09	7.2951E-04	3.3384E-11	1.4110E-09	**8.8830E-01**
F27	5.4941E-11	3.0199E-11	**4.7335E-01**	9.2603E-09	2.0338E-09	3.0199E-11	1.7769E-10	6.7650E-05
F28	3.0199E-11	3.0199E-11	1.2870E-09	8.9934E-11	3.6897E-11	3.0199E-11	3.0199E-11	1.5964E-07
F29	3.0199E-11	3.0199E-11	3.0199E-11	4.0772E-11	3.0199E-11	3.0199E-11	3.0199E-11	**8.3026E-01**
F30	3.0199E-11	3.0199E-11	3.0199E-11	3.0199E-11	3.0199E-11	3.0199E-11	3.0199E-11	7.1988E-05

**Table 7:** P-value on CEC2022 (dim = 20).

Function	GWO	WOA	PSO	AVOA	CPO	BKA	DBO	SBOA
F1	3.0199E-11	3.0199E-11	3.0199E-11	3.0199E-11	3.0199E-11	3.0199E-11	3.0199E-11	3.0199E-11
F2	1.1737E-09	3.0199E-11	3.0103E-07	1.2023E-08	7.0430E-07	5.4941E-11	7.7387E-06	8.1465E-05
F3	3.0199E-11	3.0199E-11	3.0199E-11	3.0199E-11	2.4913E-06	3.0199E-11	3.0199E-11	1.5638E-02
F4	2.4327E-05	3.0199E-11	4.5043E-11	8.1014E-10	3.6897E-11	2.3897E-08	4.5043E-11	**2.3399E-01**
F5	1.6947E-09	3.0199E-11	1.3594E-07	3.0199E-11	4.6756E-02	4.0772E-11	3.6897E-11	1.1143E-03
F6	3.0199E-11	3.0199E-11	3.0199E-11	**4.5530E-01**	3.4742E-10	2.1959E-07	5.5329E-08	9.5767E-03
F7	7.1186E-09	3.0199E-11	2.3715E-10	3.3384E-11	2.5721E-07	7.3891E-11	6.0658E-11	4.9937E-02
F8	4.9980E-09	2.8716E-10	1.9568E-10	4.9980E-09	6.7220E-10	3.1589E-10	5.0723E-10	4.0288E-02
F9	3.0199E-11	3.0199E-11	3.0199E-11	3.0199E-11	3.0199E-11	3.0199E-11	3.0199E-11	3.0939E-06
F10	8.1200E-04	5.9673E-09	6.0104E-08	2.3768E-07	4.9327E-02	2.8314E-08	1.1228E-02	**1.1882E-01**
F11	3.0199E-11	3.0199E-11	3.0199E-11	1.0315E-02	**1.5798E-01**	3.0199E-11	1.7294E-07	2.7086E-02
F12	**1.7145E-01**	3.6897E-11	2.7548E-03	4.6390E-05	1.3853E-06	3.3520E-08	3.6459E-08	1.8567E-09

As shown in the table, the data marked as highlighted is relatively sparse, and there are fewer significant differences across the three dimensions, indicating that the newly proposed ASBOA demonstrates a clear distinction from the compared metaheuristic algorithms. Therefore, ASBOA exhibits excellent overall performance among various metaheuristic algorithms, suggesting that the three strategies we introduced effectively enhance the algorithm’s convergence speed and solution accuracy, showing significant differences when compared with other algorithms.

### 4.7 Time cost comparison between ASBOA and SBOA

**Fig 9:**
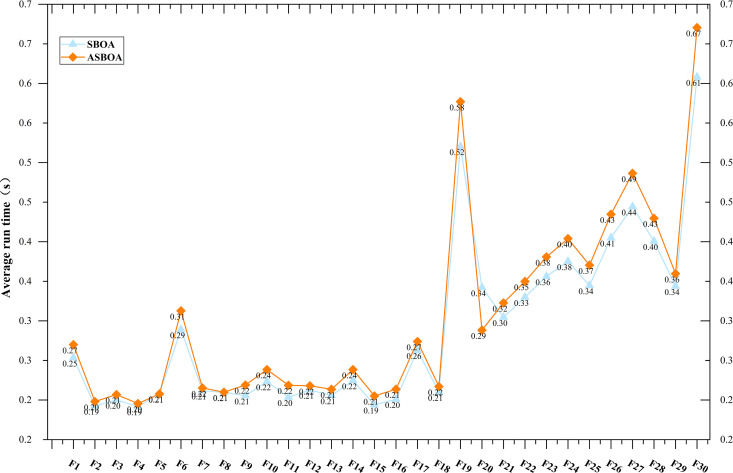
Comparison of computation time cost between ASBOA and SBOA.

Based on the previous research results, the improved ASBOA significantly outperforms the traditional SBOA in terms of overall performance. This section will focus on analyzing the differences in computational time between the two algorithms. To ensure fairness, the parameter settings for both ASBOA and SBOA are kept consistent with those used earlier. Additionally, the average runtime of the algorithms over 30 independent experiments is recorded. Fig 9 illustrates the average computational time (in seconds) required by each algorithm to solve every test function.

The experimental results on the CEC 2017 benchmark set (dim = 30) show that for unimodal functions and some relatively simple multimodal functions, the execution times of ASBOA and SBOA are essentially the same. However, when faced with more complex hybrid functions, ASBOA typically requires more computational time than SBOA. This suggests that ASBOA exhibits higher computational efficiency when tackling more complex problems. Compared to the standard SBOA, ASBOA not only employs a more efficient search strategy but also performs better in terms of global search ability and local convergence speed. Overall, ASBOA achieves higher solution accuracy than SBOA on most test functions, and the slight increase in runtime is negligible.

### 4.8 Exploration pattern analysis

In this section, the optimization performance of ASBOA during the search process is analyzed using search paths, average fitness values, search trajectories, and convergence trend plots. The convergence trend reflects the best fitness value achieved at each iteration, while the average fitness value represents the mean fitness level of all individuals at each iteration. The trajectory curve records the dynamic changes in the first dimension during the search process, and the search path intuitively presents the distribution of visited locations throughout the optimization process.

**Fig 10:**
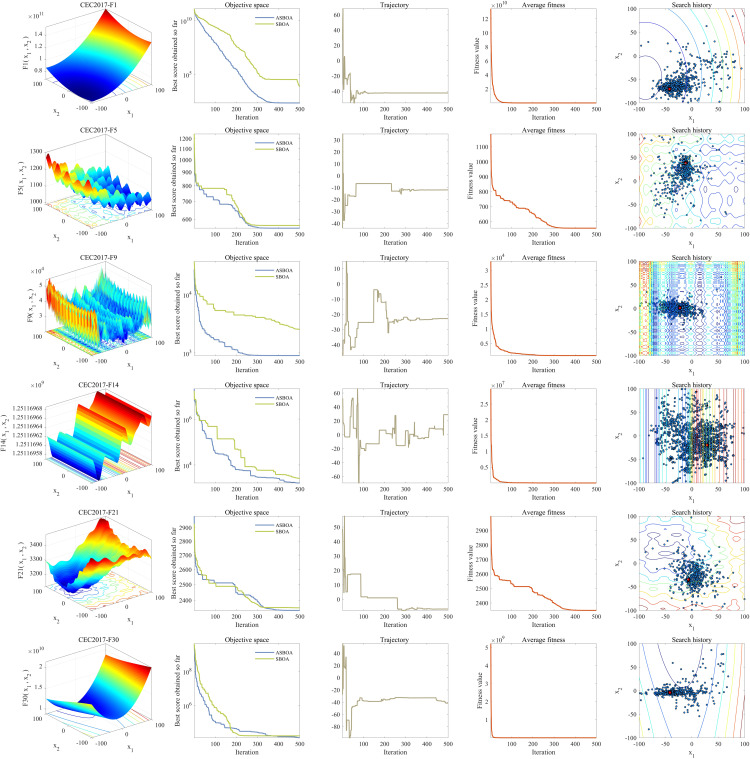
Different indexes change in ASBOA optimization process.

The second column of Fig 10 illustrates the evolution curves of the average fitness values for SBOA and ASBOA, highlighting the competitive advantage of ASBOA. Compared to SBOA, ASBOA identifies solutions progressively closer to the optimal solution of the test functions as iterations proceed. The third column depicts the search trajectories of ASBOA in the first dimension, demonstrating its ability to initially explore potential high-quality regions during the exploration phase, followed by transitioning into an exploitation phase that focuses on refining the identified superior regions to enhance solution precision.

The fourth column shows the convergence curves of ASBOA across various test functions, displaying a rapid decline trend that indicates its efficiency in approaching the optimal solutions. The fifth column visualizes the positional changes of particles during the optimization process. It can be observed that ASBOA exhibits a flexible search pattern across different stages: initially performing a comprehensive exploration of the entire search space, then gradually concentrating on smaller regions for in-depth exploitation, and ultimately locating solutions that are closest to the optimal.

### 4.9 Applied ASBOA for Wireless Sensor Network Deployment

**Fig 11:**
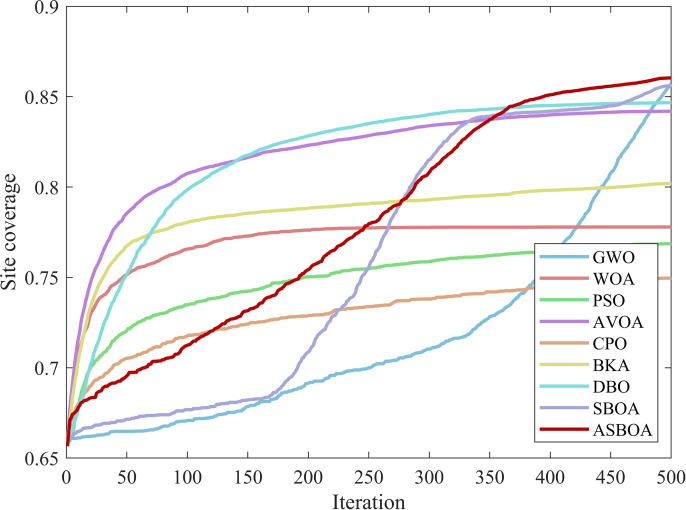
WSN convergence curve.

**Fig 12:**
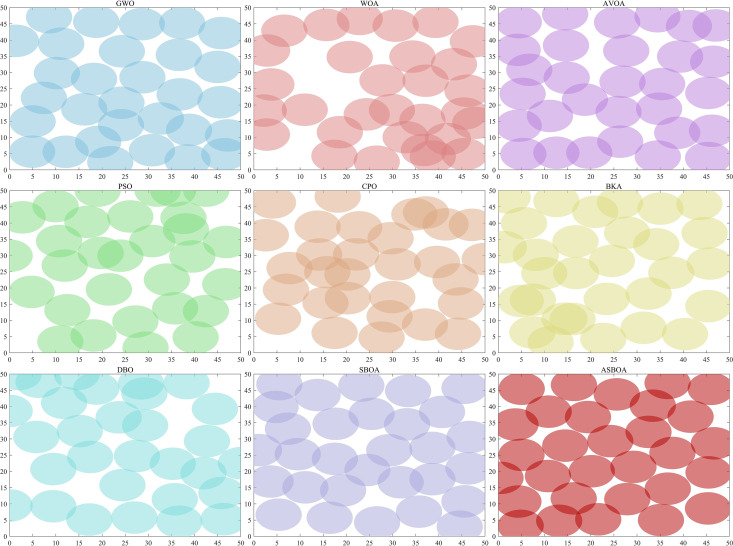
WSN optimized by different algorithms.

In the experiment, N nodes of the wireless sensor network are randomly deployed in an m×n area. Specifically, N=30 represents the number of nodes, and the area covered is m×n=50×50. The communication radius of the sensors is set to $R_{T}=5$ for one case and $R_{T}=10$ for another. To compare the performance of the algorithms, the parameters of all algorithms in the fitness function are set to be identical, as shown in Table 1. The population size for all algorithms is set to 30, and the number of iterations is set to 500. To ensure the fairness of the experiment, 30 independent runs were conducted with the same environment and parameter settings to eliminate randomness, and the relevant experimental results were recorded. The minimum (Min), maximum (Max), median (Median), mean (Mean), standard deviation (Std) and number of failed nodes (FN) for each comparison algorithm were statistically analyzed, with the optimal values highlighted in bold in Table 8. In this study, the faulty nodes are randomly selected to simulate unpredictable node failures in real-world wireless sensor networks caused by energy depletion or environmental factors. The convergence curves of different algorithms in the WSN are shown in Fig 11, and the node deployment optimized by each algorithm is shown in Fig 12.

**Table 8:** Statistics of WSN experiment results.

Algorithm	Min	Median	Max	Mean	Std	FN
GWO	81.72%	84.72%	87.08%	84.49%	1.35%	5
WOA	70.64%	75.98%	80.88%	76.09%	2.12%	7
PSO	74.12%	76.50%	79.16%	76.56%	1.27%	6
AVOA	80.44%	84.36%	86.44%	84.13%	1.66%	5
CPO	73.44%	74.86%	76.40%	75.00%	0.96%	4
BKA	73.32%	81.52%	83.44%	80.98%	2.20%	6
DBO	77.04%	82.98%	85.24%	82.73%	1.76%	6
SBOA	81.88%	85.34%	87.20%	85.27%	1.25%	4
ASBOA	**82.68%**	**85.76%**	**88.32%**	**86.47%**	**0.72%**	**2**

As observed from Table 8, the performance of the proposed Multi-strategy Gray Wolf Optimization Algorithm (ASBOA) in the Wireless Sensor Network (WSN) is significantly better than that of the other comparison algorithms in terms of the minimum (Min), maximum (Max), median (Median), and mean (Mean) values. This indicates that ASBOA achieves superior results, attaining better coverage, with a maximum coverage rate of 88.32%, compared to 87.20% for the standard SBOA. ASBOA thus demonstrates an improvement of 1.12% over SBOA. Furthermore, the average coverage rate of ASBOA is also much lower than that of the other comparison algorithms, which suggests that the proposed ASBOA exhibits strong robustness for this problem, outperforming other algorithms. In terms of the number of failed nodes, the proposed ASBOA demonstrates superior performance with only 2 failed nodes out of a total of 30 nodes. In comparison, the standard SBOA and the recently proposed CPO each have 4 failed nodes. The WOA exhibits the highest number of failed nodes at 7, followed by PSO, DBO, and BKA, each with 6 failed nodes. These results indicate that ASBOA is more suitable for WSM problems than these other algorithms. These results confirm that the proposed ASBOA demonstrates outstanding optimization performance in WSNs, providing a powerful tool for solving the wireless sensor network node deployment problem.

As shown in Fig 11, although ASBOA does not converge as quickly as GWO, WOA, BKA, AVOA, PSO, and CPO in the early iterations, it continues to improve as the number of iterations increases and ultimately achieves better results. In contrast, other algorithms become trapped in local optima and fail to find better solutions. This suggests that the proposed ASBOA has excellent potential for solving the wireless sensor network (WSN) node deployment optimization problem. As seen in Fig 12, compared to the node deployments optimized by other algorithms, the deployment distribution optimized by ASBOA is more reasonable, with less overlap and fewer blank areas. Each node is deployed more evenly. In comparison to the optimizations by other algorithms, such as WOA, CPO, and BKA, which show significant overlap of deployed nodes—an unreasonable outcome—ASBOA’s more rational deployment results in multiple benefits in terms of energy efficiency, coverage, data accuracy, network reliability, cost, and delay. This makes the entire network more sustainable, reliable, and cost-effective. From another perspective, this further validates the effectiveness of ASBOA.

### 4.10 Engineering optimization problem

#### 4.10.1 Three-bar Truss Design Problem.

**Fig 13:**
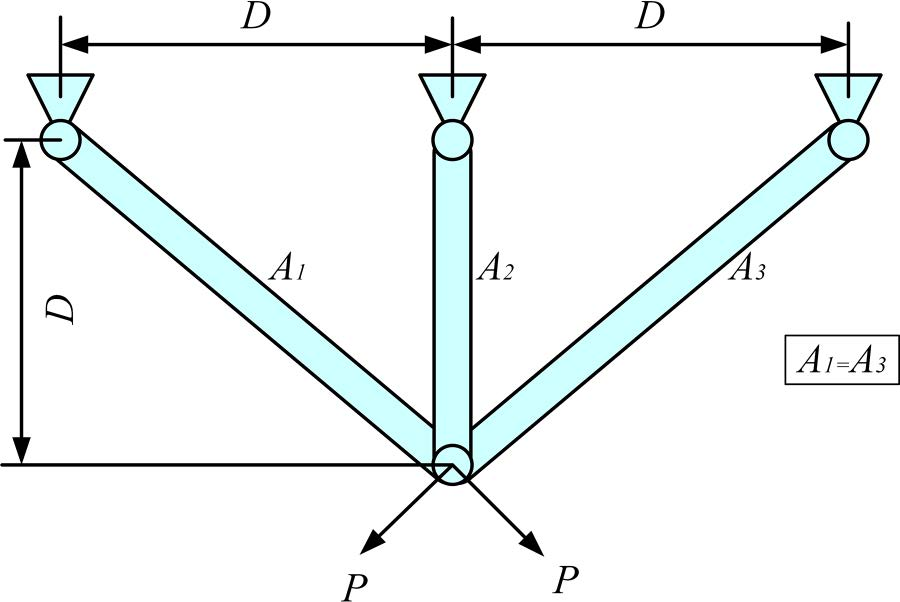
Three bar truss structure.


The three-bar truss design problem is a classic optimization problem widely applied in the field of civil engineering. The primary objective of this problem is to minimize the overall structural weight by optimizing design parameters, thereby improving material efficiency and the economic performance of the engineering structure. The design involves the adjustment of two key parameter variables, which directly affect the geometry and dimensions of the truss, ultimately determining the structure’s mass and performance. The specific structural form of the three-bar truss is shown in Fig 13. The mathematical model of this problem is given by equation (20).

$$\text{Consider }\vec{x}=\left[x_{1}\;x_{2}\right]=\left[A_{1}\;A_{2}\right],$$ (20)

$$\text{Minimize }f\left(\vec{x}\right)=l*\left(2\sqrt{2x_{1}}+x_{2}\right),$$

$$\text{\{Subject\textbackslash{} to\textbackslash{} }g{\}}_{1}(\vec{x})=\frac{\sqrt{2x_{1}}+x_{2}}{\sqrt{2x_{1}^{2}}+2x_{1}x_{2}}P-\sigma \leq 0,$$

$$g_{2}(\vec{x})=\frac{x_{2}}{\sqrt{2x_{1}^{2}}+2x_{1}x_{2}}P-\sigma \leq 0,$$

$$g_{3}(\vec{x})=\frac{1}{\sqrt{2x_{2}}+x_{1}}P-\sigma \leq 0,$$

$$\text{Parameter\textbackslash{} range\textasciitilde{}\textasciitilde{}\textasciitilde{}\textasciitilde{}\textasciitilde{}\textbackslash{} }0\leq x_{1},x_{2}\leq 1,$$

Where$l=100\text{cm},P=2\text{KN}/{\text{cm}}^{2},\sigma =2\text{KN}/{\text{cm}}^{2}.$

Table 9 presents the optimization results of ASBOA compared with other algorithms for the three-bar truss design problem. As can be seen, ASBOA achieves the best optimization result, with a value of 2.6390E + 02.

**Table 9:** Experimental results of three-bar truss design.

Algorithm	Optimal values for Variable		Optimal value	Ranking
	A_1_	A_2_		
GWO	7.8690E-01	4.1331E-01	2.6390E + 02	6
WOA	7.5929E-01	4.9842E-01	2.6460E + 02	9
PSO	7.8328E-01	4.2371E-01	2.6392E + 02	7
AVOA	7.8153E-01	4.2885E-01	2.6393E + 02	8
CPO	7.8868E-01	4.0824E-01	2.6390E + 02	2
BKA	7.8863E-01	4.0837E-01	2.6390E + 02	4
DBO	7.8973E-01	4.0528E-01	2.6390E + 02	5
SBOA	7.8871E-01	4.0814E-01	2.6390E + 02	3
**ASBOA**	**7.8868E-01**	**4.0825E-01**	**2.6390E + 02**	**1**

#### 4.10.2 Tension/Compression Spring Design Problem.

**Fig 14:**
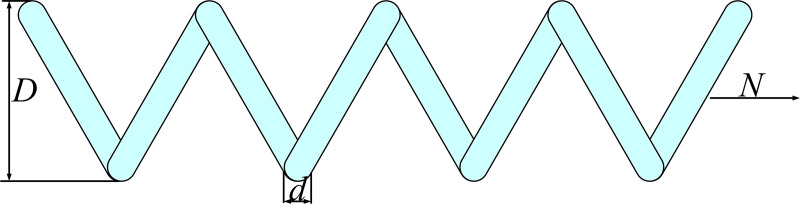
Stretch/compress spring structure.


This design problem falls within the field of mechanical design optimization, with the objective of achieving lightweight design by minimizing the weight of the tension/compression spring. The optimization task focuses on three key parameters of the spring: wire diameter (d), coil diameter (D), and number of coils (N). These parameters not only directly affect the weight of the spring but also determine its mechanical performance and service life under tension and compression conditions. By optimizing these variables in a reasonable manner, material consumption and production costs can be significantly reduced while meeting the design strength and functional requirements. The geometric structure and working principle of this engineering problem are shown in Fig 14, with the detailed mathematical description provided by equation (21).

$$\text{Consider: }\vec{x}\text{ = }[\begin{array}{ccc} x_{\text{1}} & x_{\text{2}} & x_{\text{3}} \end{array}]\text{ = }[d\;D\;N]\text{,}$$ (21)

$$\text{Minimize: }f(\vec{x})=\left(x_{\text{3}}\text{ + 2}\right)x_{\text{2}}x_{\text{1}}^{\text{2}}\text{,}$$

$$\text{Subject\textbackslash{} to: }g_{1}(\vec{x})=1-\frac{x_{2}^{3}x_{3}}{71785x_{1}^{4}}\leq 0,$$

$$g_{2}(\vec{x})=\frac{4x_{2}^{2}-x_{1}x_{2}}{12566\left(x_{2}x_{1}^{3}-x_{1}^{4}\right)}+\frac{1}{5108x_{1}^{2}}\leq 0$$

$$g_{3}(\vec{x})=1-\frac{140.45x_{1}}{x_{2}^{2}x_{3}}\leq 0,$$

$$g_{4}(\vec{x})=\frac{x_{1}+x_{2}}{1.5}-1\leq 0$$

$$\text{Parameters\textbackslash{} range}:\;0.05\leq x_{1}\leq 2,\;0.25\leq x_{2}\leq 1.3,\;2\leq x_{3}\leq 15.$$

Table 10 presents the optimization results of AGRO compared with other algorithms for the tension/compression spring design problem. As shown, the optimization results of ASBOA outperform those of the other comparison algorithms, with the optimal value being 1.2668E-02.

**Table 10:** Experimental results of tension/compression spring design.

Algorithm	Optimal values for Variable			Optimal value	Ranking
	*d*	*D*	*N*		
GWO	5.4928E-02	4.3950E-01	7.7020E + 00	1.2865E-02	6
WOA	5.5927E-02	4.6751E-01	6.8732E + 00	1.2975E-02	7
PSO	5.6118E-02	4.7291E-01	6.7312E + 00	1.3003E-02	8
AVOA	5.8874E-02	5.5546E-01	5.0321E + 00	1.3539E-02	9
CPO	5.1039E-02	3.4118E-01	1.2270E + 01	1.2683E-02	2
BKA	5.0305E-02	3.2388E-01	1.3530E + 01	1.2728E-02	4
DBO	5.0000E-02	3.1734E-01	1.4045E + 01	1.2729E-02	5
SBOA	5.0457E-02	3.2779E-01	1.3211E + 01	1.2694E-02	3
**ASBOA**	**5.1268E-02**	**3.4668E-01**	**1.1903E + 01**	**1.2668E-02**	**1**

#### 4.10.3 Cantilever beam design problem.

**Fig 15:**
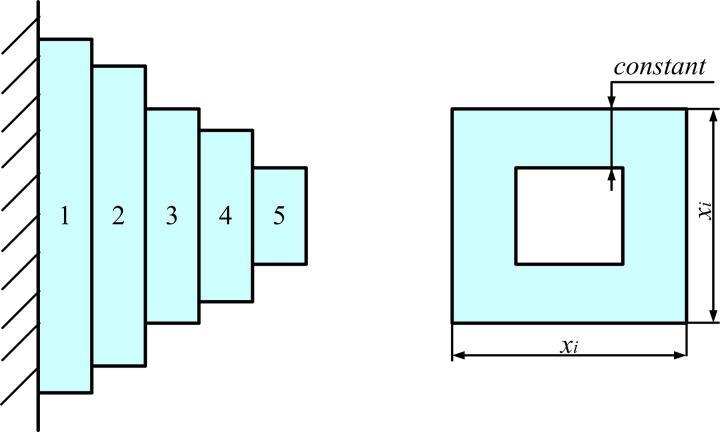
Schematic representation of Cantilever beam.


The cantilever beam design problem is a typical engineering optimization problem, with the goal of optimizing the design of a five-cube hollow structure. The core task of this problem is to adjust key parameters in such a way that the cost is minimized while meeting strength, stiffness, and other specific design constraints. The optimization process requires balancing material efficiency, manufacturing complexity, and structural performance to achieve a cost-effective design solution. The structure of this problem is shown in Fig 15, which visually illustrates the geometry and design characteristics of the cantilever beam. The specific optimization objectives and constraints are given by equation (22).

$$\text{Consider: }\vec{x}=\left[x_{1}\;x_{2\;}x_{3}\;x_{4}{\;x}_{5}\right],$$ (22)

$$\text{Minimize:\textbackslash{} }f\left(\vec{x}\right)=0.6224\left(x_{1}+x_{2}+x_{3}+x_{4}+x_{5}\right),$$

$$\text{Subject\textbackslash{} to:\textbackslash{} }g\left(\vec{x}\right)=\frac{60}{x_{1}^{3}}+\frac{27}{x_{2}^{3}}+\frac{19}{x_{3}^{3}}+\frac{7}{x_{4}^{3}}+\frac{1}{x_{5}^{3}}-1\leq 0,$$

$$\text{Parameter\textbackslash{} range: \textbackslash{} }0.01\leq x_{1},x_{2},x_{3},x_{4},x_{5}\leq 100,$$

Table 11 demonstrates the outstanding performance of ASBOA and other comparison algorithms in optimizing the CBD problem, where ASBOA achieves the best objective function value of 1.3400E + 00, outperforming the other comparison algorithms.

**Table 11:** Experimental results of cantilever beam design.

Algorithm	Optimal values for Variable					Optimal value	Ranking
	$x_{1}$	$x_{2}$	$x_{3}$	$x_{4}$	$x_{5}$		
GWO	6.0395E + 00	5.2724E + 00	4.5082E + 00	3.4785E + 00	2.1769E + 00	1.3401E + 00	6
WOA	5.6793E + 00	6.4783E + 00	4.9597E + 00	2.8060E + 00	2.5784E + 00	1.4041E + 00	9
PSO	5.9883E + 00	5.3265E + 00	4.4502E + 00	3.5465E + 00	2.1646E + 00	1.3401E + 00	7
AVOA	6.0629E + 00	5.2801E + 00	4.6998E + 00	3.3529E + 00	2.1115E + 00	1.3421E + 00	8
CPO	6.0056E + 00	5.3167E + 00	4.5116E + 00	3.4992E + 00	2.1410E + 00	1.3400E + 00	3
BKA	6.0491E + 00	5.2831E + 00	4.4832E + 00	3.5050E + 00	2.1539E + 00	1.3400E + 00	5
DBO	6.0424E + 00	5.2865E + 00	4.4893E + 00	3.5075E + 00	2.1485E + 00	1.3400E + 00	4
SBOA	6.0149E + 00	5.3012E + 00	4.4938E + 00	3.5098E + 00	2.1540E + 00	1.3400E + 00	2
**ASBOA**	**6.0109E + 00**	**5.3112E + 00**	**4.4982E + 00**	**3.5006E + 00**	**2.1528E + 00**	**1.3400E + 00**	**1**

The experimental results from the three engineering optimization design problems demonstrate that the proposed ASBOA shows significant advantages in addressing real-world engineering optimization problems. Compared to other comparison algorithms, ASBOA not only finds better solutions but also effectively reduces engineering costs, showcasing its efficiency and practical value in real-world applications.

## 5. Summary and prospect

This paper proposes an improved version of the SBOA, aimed at enhancing the convergence speed and solution accuracy of the standard SBOA, while reducing the risk of ASBOA getting trapped in local optima. First, a Differential Cooperative Search Mechanism is employed to reduce the risk of the ASBOA algorithm becoming trapped in local optima, and an Optimal Boundary Control Mechanism is used to avoid ineffective exploration, improving the convergence speed of the algorithm. Additionally, an Information Retention Control Mechanism is applied to update the population, ensuring that the current global optimal solution cannot be replaced, further enhancing the algorithm’s convergence speed.

The ASBOA algorithm is tested using the CEC2017 and CEC2022 benchmark functions. The results show that for CEC2017 (dimensions = 30 and 100) and CEC2022(dimensions = 20), ASBOA achieves the best ranking in 76.67% (23 out of 30), 86.67% (26 out of 30) and 75% (9 out of 12) of the functions, respectively. It is also applied to the WSN problem and three engineering problems, where it outperforms all comparison algorithms. In the WSN problem, the coverage rate can reach 88.32%, an improvement of 1.12% over the standard SBOA. These results demonstrate that the proposed ASBOA has strong overall performance and holds great promise for optimization applications.

Experimental observations on the CEC2017 and CEC2022 test set show that ASBOA can quickly converge to a global optimal solution in most cases. However, there is still a risk of getting trapped in local optima (e.g., in CEC2017-F26, F27, and F29, and CEC2022-F10). Furthermore, the coverage rate in the WSN problem remains insufficient and requires further improvement. Due to a lack of population diversity, stagnation may occur. Other potential issues still need to be further tested and validated.

Although ASBOA has demonstrated excellent performance in wireless sensor network (WSN) node deployment optimization and engineering optimization problems, there remain areas for improvement. First, while ASBOA outperforms other algorithms in terms of coverage and optimization results, its convergence speed in the early stages is relatively slow, which may reduce its efficiency in real-world applications requiring rapid responses. Second, although ASBOA exhibits strong robustness, its performance and stability under larger-scale problems or more complex constraints have not been fully validated. Additionally, the sensitivity of its parameters and its adaptability to different problems require further investigation.

Future research can focus on several promising directions. First, the introduction of dynamic parameter adjustment mechanisms or more efficient search strategies could enhance the algorithm’s convergence speed during early iterations, allowing it to achieve high-quality solutions faster. For larger-scale networks or problems with complex constraints, designing more adaptive strategies or hybrid algorithms will be essential to improve the method’s flexibility and broaden its applicability. At the same time, efforts to optimize the algorithm’s structure could significantly reduce computational complexity, making it more suitable for applications requiring high real-time responsiveness. Another intriguing direction lies in exploring the integration of machine learning or deep learning techniques, which can leverage data-driven approaches to optimize parameter settings or improve the modeling of complex problems. Furthermore, in-depth theoretical analyses are needed to better understand the algorithm’s convergence properties and computational complexity, along with the development of automated parameter adjustment methods to minimize the need for manual tuning. Additionally, future work may extend to the design of deployment strategies specifically for wireless sensor networks (WSNs), addressing challenges such as k-coverage and k-connectivity while simultaneously minimizing network costs and maximizing network lifespan. Developing multi-objective and binary versions of ASBOA could also expand its capability to meet diverse problem requirements. These advancements collectively hold the potential to further enhance the efficiency and versatility of ASBOA, establishing it as a robust and general-purpose tool for tackling complex optimization challenges.
